# Lost in translation: the lack of agreement between surgeons and scientists regarding biomaterials research and innovation for treating bone defects

**DOI:** 10.1186/s12916-024-03734-z

**Published:** 2024-11-06

**Authors:** Markus Laubach, Stephen Whyte, Ho Fai Chan, Tina Frankenbach-Désor, Susanne Mayer-Wagner, Frank Hildebrand, Boris M. Holzapfel, Ulrich Kneser, Uwe Dulleck, Dietmar W. Hutmacher

**Affiliations:** 1 Department of Orthopaedics and Trauma Surgery, Musculoskeletal University Center Munich (MUM), LMU University Hospital, LMU Munich, Munich, Germany; 2 Australian Research Council (ARC) Training Centre for Multiscale 3D Imaging, Modelling, and Manufacturing (M3D Innovation), Queensland University of Technology, Brisbane, QLD 4000 Australia; 3 School of Economics and Finance, Queensland University of Technology (QUT), 2 George St, Brisbane, QLD 4001 Australia; 4 Centre for Behavioural Economics, Society & Technology (BEST), Queensland University of Technology (QUT), Brisbane, QLD 4001 Australia; 5 ARC Training Centre for Cell and Tissue Engineering Technologies, Queensland University of Technology (QUT), Brisbane, QLD 4059 Australia; 6 ARC Training Centre for Behavioural Insights for Technology Adoption, Queensland University of Technology (QUT), Brisbane, QLD 4001 Australia; 7 Department of Orthopaedics, Trauma and Reconstructive Surgery, RWTH Aachen University Hospital, Pauwelsstraße 30, 52074 Aachen, Germany; 8 Department of Hand, Plastic, and Reconstructive Surgery, BG Trauma Center Ludwigshafen, Heidelberg University, Ludwigshafen, Germany; 9 Faculty of Business Government and Law, University of Canberra, Canberra, Australia; 10 ARC Training Centre in Additive Biomanufacturing, Queensland University of Technology, Brisbane, QLD 4059 Australia; 11 Max Planck Queensland Centre (MPQC) for the Materials Science of Extracellular Matrices, Queensland University of Technology, Brisbane, QLD 4000 Australia

**Keywords:** Tissue engineering, 3D printing, Interdisciplinary communication, Bone substitutes, Survey study

## Abstract

**Background:**

With over 2 million grafts performed annually, bone ranks second only to blood in the frequency of transplants. This high demand is primarily driven by the persistent challenges posed by bone defects, particularly following trauma or surgical interventions such as tumour excision. The demand for effective and efficient treatments has increased exponentially in the twenty-first century. Limitations associated with autologous bone grafts drive exploration into replacements, including allografts, synthetic substitutes, and 3D-printed scaffolds. This research aimed to unravel disparities in the knowledge and evaluation of current and future bone defect treatments between surgeons and biomaterial scientists.

**Methods:**

A prospective cross-sectional survey, pre-registered with the OSF (https://osf.io/y837m/?view_only=fab29e24df4f4adf897353ac70aa3361) and conducted online from October 2022 to March 2023, collected data on surgeons’ views (*n* = 337) and scientists (*n* = 99) on bone defect treatments.

**Results:**

Scientists were significantly more optimistic than surgeons regarding the future replacement of autologous bone grafts with synthetic or tissue-engineered substitutes (*p* < 0.001). Accordingly, scientists foresee a paradigm shift from autologous bone grafts to biomaterial and tissue-engineered solutions, reflecting their confidence in the ongoing advancements within this field.

Furthermore, regulatory trepidations for 3D-printed bone scaffolds were acknowledged, with scientists emphasizing the need for a more significant focus on clinical relevance in preclinical studies and regulatory clarity. In a ranked categorical assessment, witnessing the technology in action was deemed most influential in adopting new bone regeneration methods by both scientists and surgeons.

**Conclusions:**

To conclude, this study was conducted through a web-based survey, highlighting a substantial translational gap. It underscores the immediate need (“call to action”) for meaningful interdisciplinary collaboration between surgeons and scientists, often referred to as the need to “walk the talk”. The findings underscore the critical importance of aligning clinical needs, research outcomes, and regulatory frameworks to improve the development and implementation of biomaterial-based bone graft substitutes that demonstrate efficacy and efficiency in bone defect treatment.

**Supplementary Information:**

The online version contains supplementary material available at 10.1186/s12916-024-03734-z.

## Background

Bone defects following trauma, (bony) infection or tumour resection still pose relevant challenges for patients, surgeons, and scientists in the twenty-first century [1–3]. Globally, 2 million bone transplants are performed every year (half a million of them in the United States of America, USA), which makes it the gold standard treatment for bone defects. This makes bone the second most frequently transplanted tissue after blood transfusions [4, 5]. However, there is relevant morbidity associated with autologous bone graft harvesting, such as iatrogenic fractures and perioperative blood loss [6]. Additionally, some patients have limited quantity or quality of autologous bone material [7, 8]. The stand-alone use of autologous bone grafts for reconstructing larger bone defects is associated with an increased risk of graft resorption [9] and results in structurally and functionally compromised regenerated bone in the reconstructed bone area (segment) [10]. These limitations necessitate the development of alternative bone substitutes. In response to these challenges, there has been growing interest in the use of natural and/or synthetic bone substitutes. Nowadays, in addition to autologous bone grafts, various bone replacement products (allografts, synthetic bone replacement materials, growth factors and bioactive molecules) along with artificial 3D-printed scaffolds, known as bone scaffolds, are used for bone defect regeneration [11].

In 1965, Urist [12] described an osteoinductive biomaterial for the first time in the preparation of soluble extracts of demineralized bone (demineralized bone matrix, DBM) derived from allograft. Since this groundbreaking work, numerous preclinical animal studies have confirmed the efficacy of DBM in human medicine. While the utilization of allografts is on the rise, owing to their immediate structural support and osteoconductivity, natural bone substitutes, including allografts, exhibit highly variable bone regenerative (osteoinductive) properties, increased rates of non-union, and the potential for disease transmission [13, 14]. In addition, fractures of allografts in major bone defects [15] and their high cost have further encouraged the development of other strategies, including the use of synthetic bone substitutes [16]. Synthetic bone substitutes such as hydroxyapatite (HA) and/or tricalcium phosphate (TCP) can only be used in combination with autologous bone grafts in a maximum ratio of 1:1 to 1:3, as they have a high rate of non-unions when used alone as a bone substitute [13, 17–20]. Therefore (as with allografts), the clinical applicability of synthetic bone substitutes for bone defects is limited. Three-dimensional printing (3D printing) is a form of additive manufacturing wherein objects are produced in successive layers from digital models [21]. Patient-specific 3D-printed bone scaffolds can be made from macroporous (biodegradable) bioceramics (e.g. HA) [22], non-resorbable porous titanium (e.g. Ti6Al4V) [23–25], and biodegradable composite materials such as medical-grade polycaprolactone and tricalcium phosphate (mPCL-TCP) [1, 26, 27]. Surgical strategies applying patient-specific scaffold-guided bone regeneration (SGBR) offer the capacity to regenerate bony defects [28].

The latest advancements in automation, speed, reproducibility, and flexibility with small batches, coupled with the potential for reduced manufacturing costs, make the areas of bone replacement products and 3D printing technologies for orthopaedic trauma surgery very attractive in principle. These innovations lay a strong foundation for the successful transition from laboratory research to clinical practice [29–32]. Over the past two decades, bioengineering and biotechnology have witnessed an exponential increase in publications. Yet, this surge in scientific knowledge has not been matched by a corresponding increase in clinical implementation that directly benefit patient care [33]. Consequently, many discoveries fail to translate into U.S. Food and Drug Administration (FDA)-approved devices and are even less frequently adopted by the medical community [21, 34, 35]. Government bodies and regulatory authorities have begun to address the entrepreneurial challenges—previously identified as critical components of the “valley of death” some 15 years ago [36]—that impede the translation of bone graft substitutes and 3D-printed bone scaffolds into clinical applications. These efforts are aimed at bolstering the research and industrial landscapes: (1) regulatory approval in many countries has been negotiated and patient-oriented solutions have been found, and (2) increased allocation of third-party funding for the financing of confirmatory large animal studies [37, 38] and clinical studies [39] has become more common. Despite overcoming the stony path of research, development, and regulatory approval, there is no guarantee that a product will achieve clinical success or become widely adopted for routine clinical use. Key influencing factors, such as the decision-making processes of surgeons and scientists, must be thoroughly understood before widespread clinical integration can be achieved [21, 40].

In his 1959 lecture, “The Two Cultures” [41], Charles Percy Snow identified a lack of dialogue across disciplinary boundaries—an issue that persists today. Despite the highest levels of academic training in diverse fields, distinct intellectual cultures, each with its own specialized vocabulary and modes of reasoning, still hinder effective cross-disciplinary mediation [42]. Surgeons and scientists possess domain-specific expertise shaped by their academic and practical experiences. The nature of their daily work is equally specialized, and they frequently engage with healthcare professionals from varying fields and commercial stakeholders, each bringing different conceptual and scientific perspectives [43]. These challenges arose around the middle of the last century, because until around the 1950s and 1960s, basic research and clinical research in institutions were relatively closely interconnected [44]. Medical research, for example, was primarily carried out by medical scientists who also treated patients. This changed with the significant increase and specialization, especially in the field of molecular biology in the 1970s. Clinical and basic research began to separate, and biomedical research developed into an independent discipline with its own training.

At the start of the twenty-first century, interdisciplinarity emerged as a prominent topic, with many departments, research institutes, and grant funding agencies emphasizing “interdisciplinarity” in their vision statements and programs [45, 46]. Yet, in 2024, one must conclude from literature and other public resources that most biomedical research today is carried out by highly specialized scientists with PhDs, with medical doctors being in the minority [44]. Notably, the cultural and academic identities between clinicians and scientists differ [35]. Therefore, to address the reasons for this very costly translational gap and to achieve more efficient biomaterial and implant development for bone defect treatment, we have addressed the question of whether (orthopaedic, trauma, and plastic) surgeons (group: surgeons) and basic (biomaterial) scientists (group: scientists) evaluate the current options and future possibilities for the treatment of bone defects differently.

## Methods

A prospective website-based cross-sectional survey was conducted using electronic questionnaires. Surgeons and scientists were recruited via network contacts and conference meetings (convenience sampling method) and invited to complete an online survey. There was no specific method to exclude repeated participation, but the analysis of the IP addresses of the computers used by the participants revealed no evidence of multiple entries. All data was collected anonymously between October 22, 2022, and March 13, 2023, and analysed in aggregated form so that it cannot be traced back to any participant. Participants were incentivized with a chance to enter a prize draw of two cash prizes worth USD 500. The survey also collected further data that was not used in the current study (see also Ref. [47]). The entire project was conducted under the Queensland University of Technology Ethics Committee (University Human Research Ethics Clearance no. LR 2022–6352-11,321). The study was also pre-registered at Open Science Framework (OSF, https://osf.io/y837m/?view_only=fab29e24df4f4adf897353ac70aa3361), a free, open-source platform designed to support researchers in managing, sharing, and collaborating on research projects across various disciplines, promoting transparency and reproducibility in science.

### Questionnaire

The data collection included in total six sections focusing on demographic data and questions about the participants’ professional background (four questions), experience with bone defect treatment including biomaterials (four questions), availability of options and treatment concepts for bone defects (two questions), options and development of autologous bone grafts and bone substitutes (two questions), medico-legal aspects of 3D-printed medical devices for bone defect treatment (four questions), and evaluation of what is the most important aspect in deciding to use the new technology (one question). The complete questionnaire can be found in Supplement 1, and the questions in the respective sections are briefly outlined below.

#### Demographic data and occupational background

Age was asked at the beginning of the questionnaire. Furthermore, the sex could be specified subsequently, with the option of not specifying a sex. Participants were also asked to indicate the occupational category that best described their current job, with the options of (orthopaedic, trauma and plastic) surgeon and (biomaterial) scientist available as a pre-selection and a text field for free text. The country where the participants worked at the time of the survey was then queried.

#### Experience with bone defect treatment, including biomaterials

Participants were asked about their professional experience in treating bone defects in years, as well as their experience with the corresponding biomaterials for treating bone defects. Furthermore, the participants were asked to indicate the average number of surgical procedures for the treatment of bone defects that they participated in per month and the average monthly frequency of participation in interdisciplinary patient consultations with bone defects.

#### Availability of options and treatment concepts for bone defects

Participants were then asked whether they thought that the current options for surgical treatment of bone defects were sufficient on a scale of 0 to 100%. Furthermore, the participants were asked whether they agreed on a 0 to 100% scale that the available treatment guidelines for patients with bone defects are sufficiently standardized.

#### Options and development of autologous bone grafts and bone substitutes

On a scale from 0% (no agreement) to 100% (full agreement), participants were asked to give their assessment of the statement that autologous bone grafts will be replaced by bone graft substitutes in the future. In the next question, participants were asked to use the same scale to assess the extent to which they believe the developments and clinical tests for bone replacement products for the treatment of bone defects are promising.

#### Regulatory and medico-legal challenges for 3D-printed medical devices for bone defect treatment

On a scale of 0% (disagree) to 100% (strongly agree), participants indicated how much they agreed with the statement that more large animal studies, and in a subsequent question, that more randomized trials are needed before 3D-printed bone scaffolds become routine, standardized clinical use. Participants were also asked to what extent, on a scale of 0 to 100%, they agree that greater clarity is needed regarding the legal and regulatory aspects of the (clinical) use of 3D-printed bone scaffolds.

In the next question, respondents were asked to indicate which medical device laws are currently in place for 3D-printed medical devices in their healthcare system, selecting one of the following five options: (1) None—3D printing is such a new technology that it has not been regulated; (2) As they are medical products, the usual regulatory regime for medical products will apply; (3) None. 3D-printed products are custom-made and therefore excluded from the current law; (4) The 3D-printed product itself is not subject to the law, but the materials that it is made from must comply with materials standards and have to be cleared be the FDA or another regulatory body; (5) I am not certain/ sure at this time.

#### Evaluation of what is the most important aspect in deciding to use the new technology

In the following categorical ranking question [48], participants were asked to rank (1–5) a number of possible responses to the question, “If a new bone regeneration technology was introduced into clinical practice, what would most likely encourage you to switch to this new technology?”: (1) Hearing from colleagues that they have successfully performed the procedure; (2) Reading about it in a peer-reviewed medical journal; (3) Seeing that the procedure is now endorsed or recommended by the appropriate surgical society or authority; (4) Witnessing the procedure being performed online or via video link; (5) Witnessing the procedure being performed in person.

### Statistical analysis

Non-responses to individual questions were considered missing data and were therefore not included in the statistical analysis. Statistical analyses were performed using STATA/MP version 17 (StataCorp), with statistical differences between surgeons and scientists assessed using Student’s *t* test. A Pearson chi-square test (*χ*^2^ test) was used to compare categorical variables. A significance level of *p* < 0.05 was selected. The data are shown in the figures as mean value with SD (standard deviation) ( ±) or with mean value and 95% confidence interval (error bars).

## Results

### Demographic data and occupational background

Our study’s sample comprised 451 people, and the composition was differentiated according to speciality: surgeons (*n* = 337; 74.72%) and scientists (*n* = 99; 21.95%); 15 participants (3.33%) were excluded as they could not be assigned to either speciality (Table 1).

**Table 1:** Detailed occupation classification of survey participants. Please note that out of the selected choice “Other”, seven participants were allocated to a group of surgeons based on their free text response, and 15 participants could not be assigned to the group of surgeons or scientists and were therefore excluded

Occupation	*N*	Percentage (%)
Surgeon (orthopaedic)	146	32.37
Surgeon (trauma)	177	39.25
Surgeon (plastic)	7	1.55
Surgeon (other)	7	1.55
Scientist (biomaterials)	43	9.53
Scientist	56	12.42
Excluded (no surgeon or scientist)	15	3.33
**Total**	**451**	**100.00**

The sample population had a highly skewed sex ratio, with men (88.13% surgeons and 52.53% scientists) dominating over women (11.28% female surgeons and 33.38% female scientists) (*p* < 0.001), with a total of *n* = 11 participants not specifying their sex. Female surgeons were significantly older than female scientists (49.2 ± 12.0 years and 41.6 ± 11.1 years, respectively; *p* < 0.001).

Geographically, the majority of participants were based in Germany (*n* = 251 surgeons, *n* = 33 scientists), followed by the USA (*n* = 16 surgeons, *n* = 12 scientists), Switzerland (*n* = 12 surgeons, *n* = 6 scientists) and Australia (*n* = 3 surgeons, *n* = 13 scientists). The professional residence was divided into countries of the Global South and Global North, according to the usual definition of the DAC (Development Assistance Committee) list, which shows the receipt of official development assistance by OECD (Organization for Economic Co-operation and Development) countries (so-called “DAC List of ODA Recipients for reporting on aid in 2022 and 2023”) [49]. It was found that almost all participants (96.28%) work in countries in the Global North, with 3.02% of participants not providing any information.

The results of the surveys are presented below; the number of received responses per question can be found in Supplement 2.

### Experience with bone defect treatment including biomaterials

Compared to scientists, surgeons had more experience with bone defect treatment (15.1 ± 10.6 years and 9.1 ± 8.3 years, respectively; *p* < 0.001), whereas no difference was found concerning experience with biomaterials (11.5 ± 10.6 years and 12.1 ± 10.2 years, respectively; *p* = 0.637). Surgeons were significantly more likely than scientists to participate in surgical procedures for treating bone defects (4.1 ± 4.5 × per month and 1.28 ± 3.0 × per month, respectively, *p* < 0.001). There was no difference between surgeons and scientists in the number of interdisciplinary meetings to discuss case histories of patients with bone defects (2.1 ± 3.3 × per month and 1.91 ± 3.4 × per month, respectively, *p* = 0.717).

### Availability of options and treatment concepts for bone defects

**Fig. 1:**
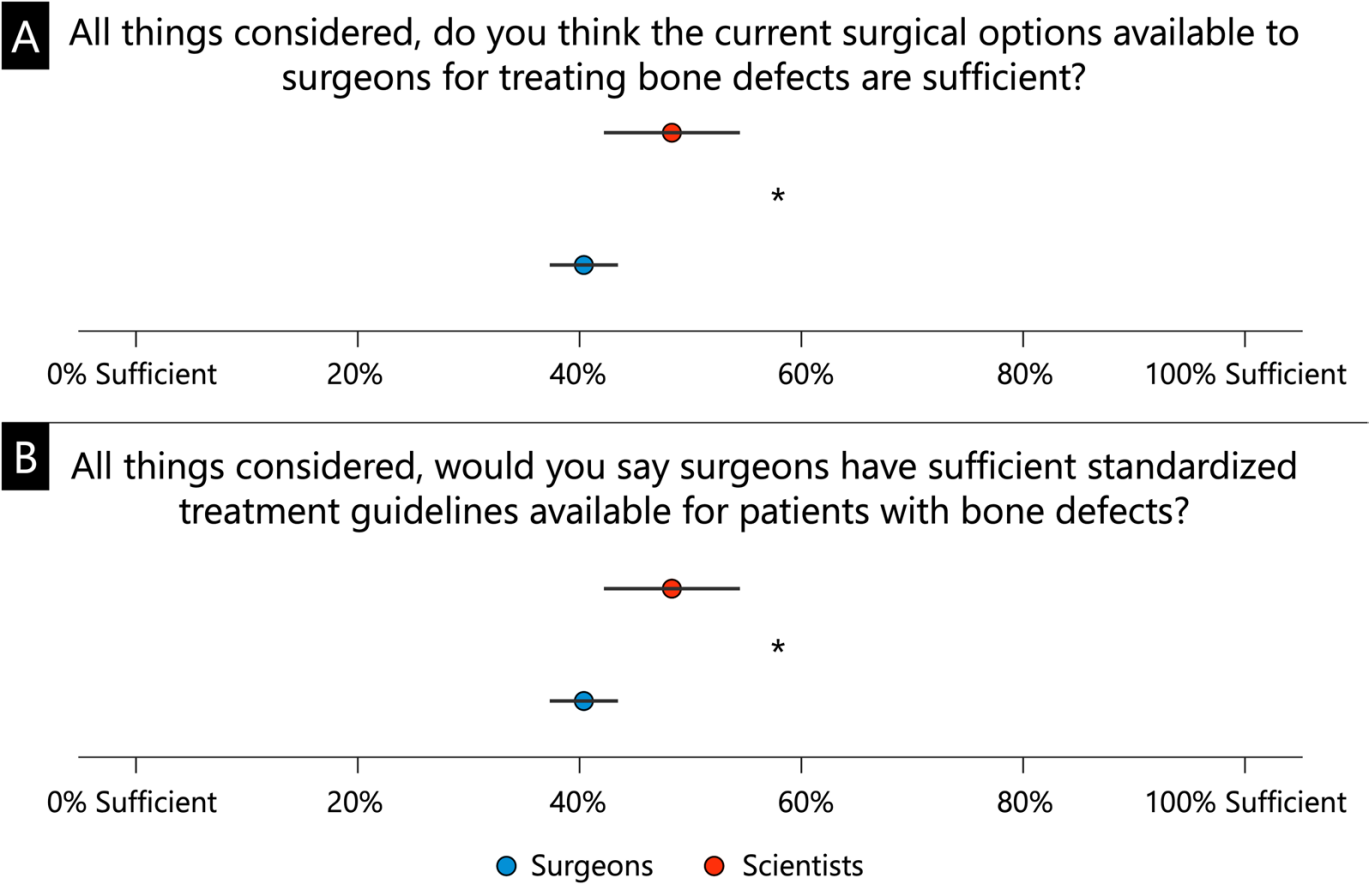
Assessment of whether sufficient options (**A**) and standardized treatment guidelines (**B**) are available for the surgical treatment of bone defects. * *p* < 0.05

Regarding the assessment of whether the current surgical options available to surgeons for treating bone defects are sufficient, significantly more surgeons were of the opinion that this was the case (54.9 ± 24.1% and 46.8 ± 23.0%, respectively; *p* = 0.013, Fig. 1A), whereas significantly fewer were of the opinion that there are sufficient standardized treatment guidelines for the treatment of bone defects (40.4 ± 24.8% and 48.3 ± 25.3%, respectively; *p* = 0.020, Fig. 1B).

### Options and development of autologous bone grafts and bone substitutes

**Fig. 2:**
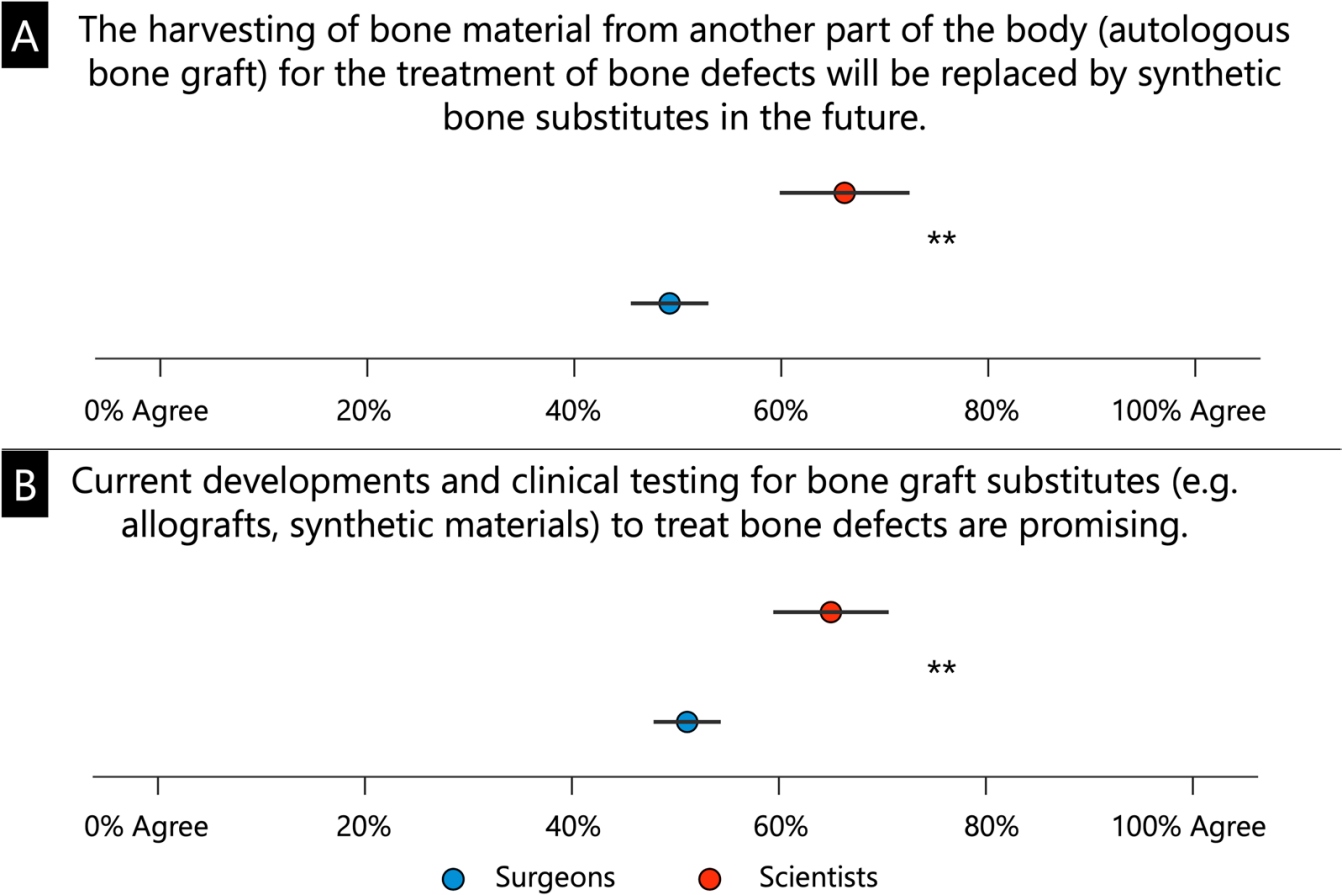
Assessment of the extent to which autologous bone grafts for the treatment of bone defects will be replaced by bone substitute products in the future (**A**), and whether the current development and clinical testing of bone substitute products is considered promising (**B**). ** *p* < 0.001

Scientists compared to surgeons were significantly more likely to believe that autologous bone grafts will be replaced by bone substitutes in the future (66.1 ± 25.3% and 49.2 ± 29.9%, respectively; *p* < 0.001, Fig. 2A) and that their development and clinical testing is promising (65.0 ± 22.5% and 51.1 ± 25.9%, respectively; *p* < 0.001, Fig. 2B).

### Regulatory and medico-legal challenges for 3D-printed medical devices for bone defect treatment

**Fig. 3:**
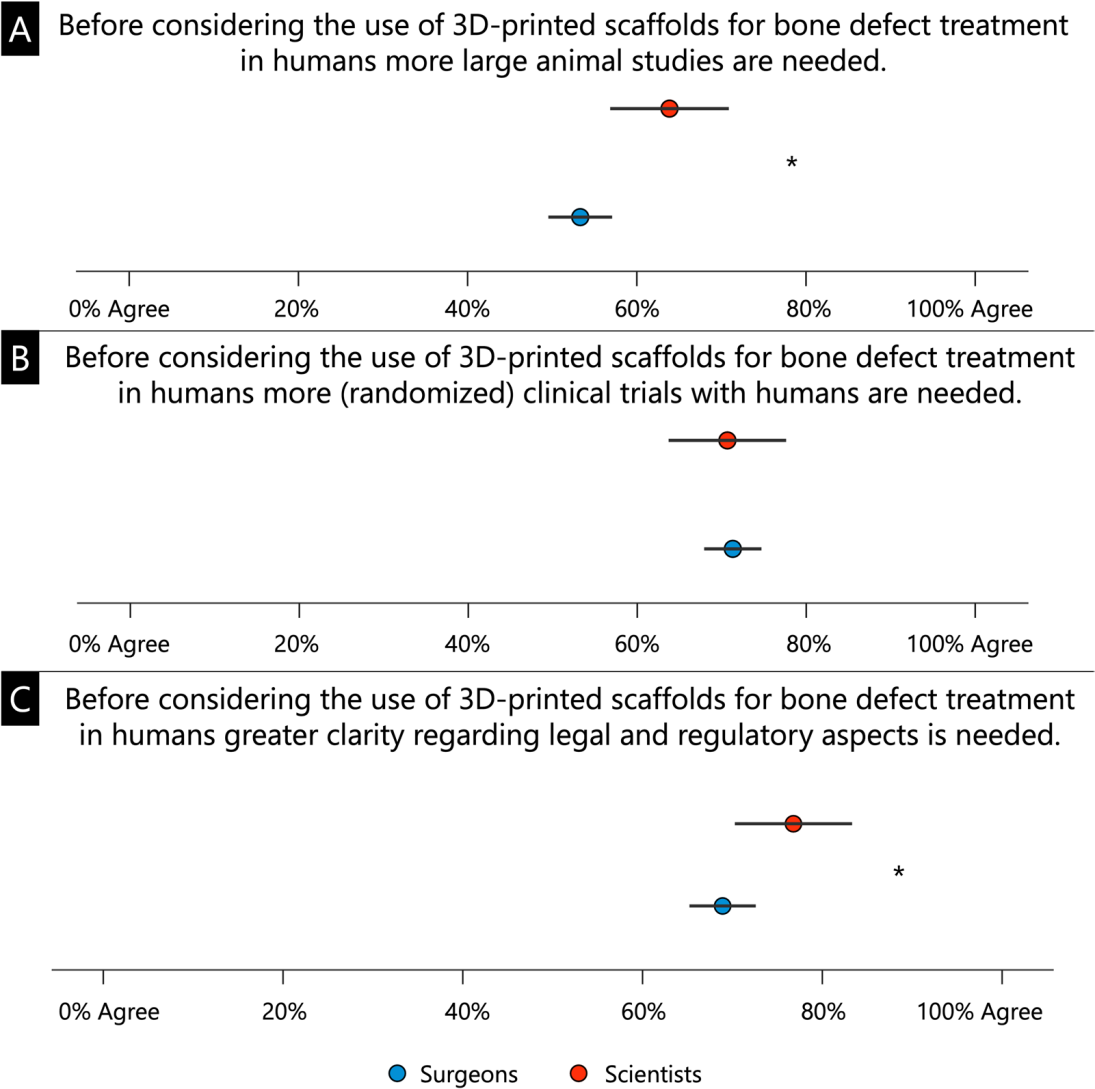
Survey results regarding the assessment of the need for (further) large animal studies for 3D-printed bone scaffolds (**A**), (randomized) clinical trials for 3D-printed bone scaffolds (**B**) and more clarity on legal and regulatory aspects (**C**). * *p* < 0.05

Regarding 3D-printed bone scaffolds for the treatment of bone defects, scientists were significantly more likely than surgeons to agree that more large animal studies are needed (63.9 ± 28.5% and 53.3 ± 30.2%, respectively; *p* = 0.011, Fig. 3A). Both groups of surgeons and scientists very often agreed that more clinical trials are needed (70.7 ± 29.3% and 71.3 ± 27.1%, respectively; *p* = 0.866, Fig. 3B). Scientists were more likely than surgeons to agree that more clarity is needed on legal and regulatory issues (76.79 ± 26.5% and 68.9 ± 29.4% respectively; *p* = 0.049, Fig. 3C).

**Fig. 4:**
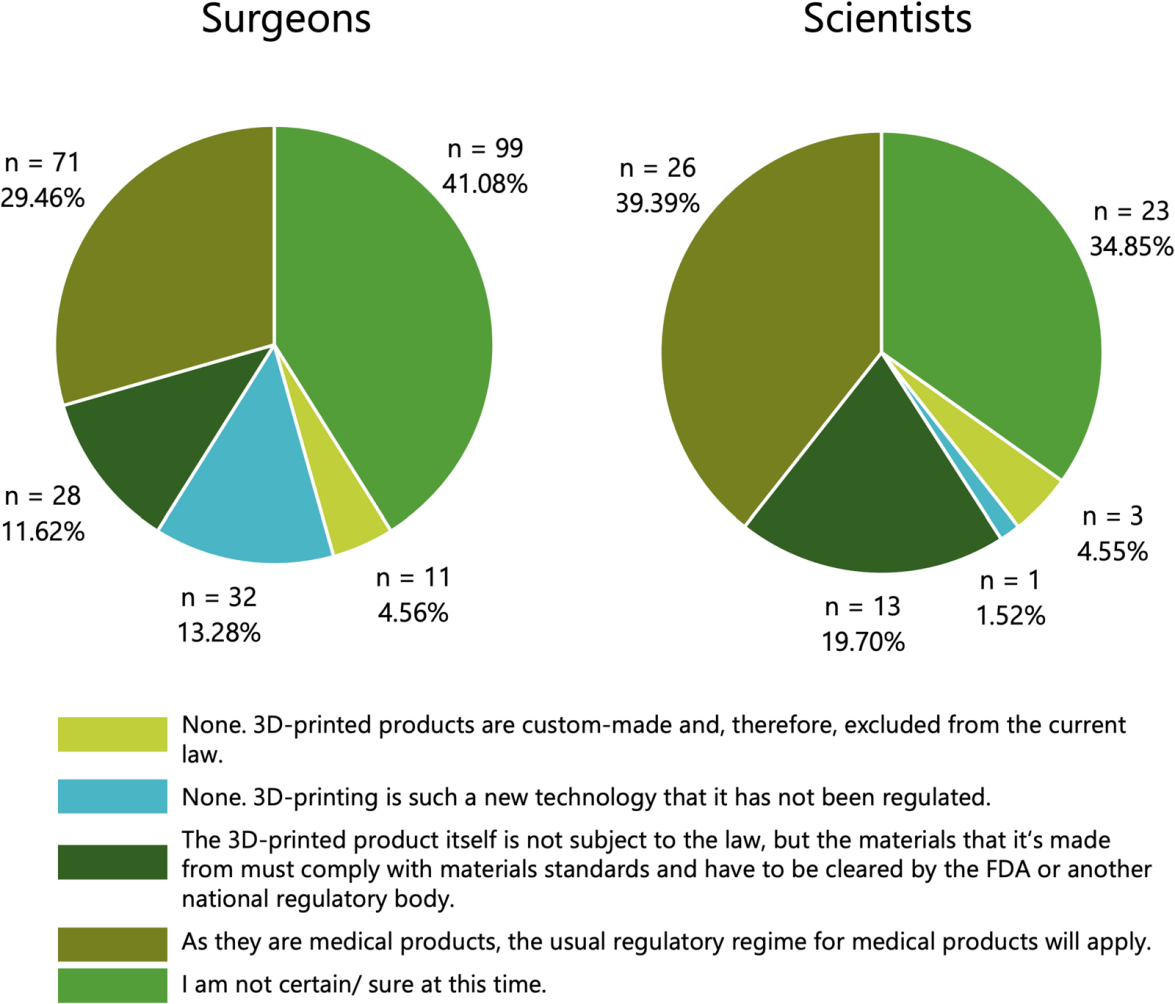
Surgeons’ and scientists’ assumptions about medical device laws for 3D-printed medical devices in their respective healthcare systems

There was a significant difference between surgeons and scientists on the question of what type of medical device laws apply to 3D-printed medical devices (*p* = 0.023, Fig. 4). It is particularly noteworthy that over a third of participants in both groups are unsure which medical device laws apply to 3D-printed implants, with 41.1% of surgeons in particular stating that they were unable to assess the legal situation (Fig. 4).

### Evaluation of what is the most important aspect in deciding to use the new technology

**Fig. 5:**
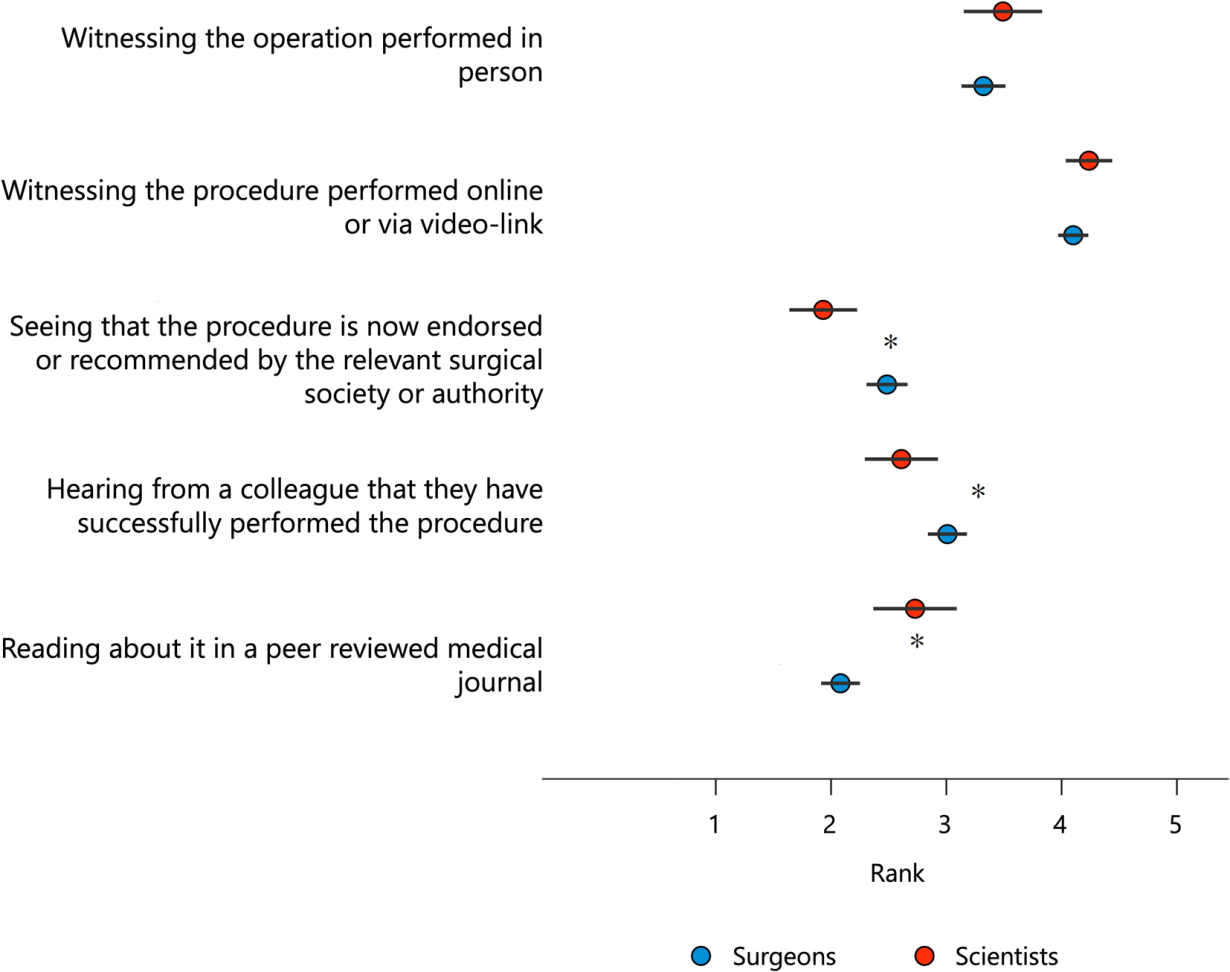
Ranking of the likelihood that the transition to a new bone regeneration technology will be introduced into clinical practice. * *p* < 0.05

When asked to rank what would most likely lead to the introduction of this new bone regeneration technology into clinical practice, witnessing the procedure either in person or online was ranked highest for both surgeons and scientists (4.10 ± 0.96% and 4.22 ± 0.77%, respectively; Fig. 5). Reading about it in a peer-reviewed medical journal was ranked lowest compared to the other answer options by surgeons (surgeons 2.10 ± 1.24% and scientists 2.71 ± 1.39%, *p* = 0.001). Scientists, on the other hand, ranked “Seeing that the procedure is now endorsed or recommended by the appropriate surgical society or authority” lowest compared with other options (surgeons 2.48 ± 1.30% and scientists 1.91 ± 1.13%, *p* = 0.003).

## Discussion

The treatment of bone defects remains a clinical challenge that is associated with high reintervention rates, high patient morbidity, and extremely high associated healthcare costs [50]. Surgical techniques, new materials for bone graft substitutes, and, more recently, 3D-printed bone scaffolds—such as the SGBR concept [1]—are constantly evolving. Since 1987, when the term tissue engineering was officially coined [51], more than 50,000 studies have been published on scaffolds, with over 40% focusing on bone tissue engineering [52]. This growth reflects the maturation of the biomaterials science field over the past four decades. Yet, even in 2024, only a tiny fraction of these innovations has been integrated into routine clinical practice, resulting in an expensive translational gap, especially in orthopaedics [53, 54]. We hypothesized that this costly disparity within the translational realm, often referred to as “from bench to bedside” [55] is attributable to the differing perspectives between surgeons and scientists regarding state-of-the-art and the innovation potential of emerging options, particularly (3D-printed) biomaterials for bone defects.

In the present web-based survey study, non-probability sampling was used, i.e. a random sample in which the participants were included without specific probabilities (so-called “convenience sampling method”) [56]. Non-probability sampling is the most commonly used type of sampling in survey statistics, especially in survey studies, as it is relatively inexpensive to conduct and relevant sample sizes can be achieved with reasonable effort [57–60]. A large sample of 337 surgeons and 99 scientists with extensive experience in the treatment of bone defects and in working with biomaterials were included in the study. This achieved sample size, coupled with participants’ significant experience, ensured sufficient “power” for valid interpretations based on the results [61]. Noteworthy, a similarly large number of scientists compared to surgeons might have shown more precise results of hitherto statistically insignificant trends. Still, this assumption is somewhat speculative and may have to be investigated in future work. It is important to note the heavily skewed sex ratio and geographical distribution of participants, with male participants from countries in the Global North dominating both groups, which should be considered when interpreting the results.

Undoubtedly, success in the laboratory is a critical factor for justifying further animal and human studies and, ultimately, routine translation into clinical practice. However, maximizing the success of laboratory experiments and research outcomes requires leveraging collaboration between research and clinic, which must be more closely integrated to be truly effective [35, 45, 62]. Clinical relevance, defined indications, and efficacy measurements must be considered, particularly in the idea generation and concept development phase, to develop a structured approach designed for successful translation from the outset [63]. The results of the survey study have shown that scientists, in particular, are less likely to attend interdisciplinary meetings, which are regularly held to discuss patients with complex bone defects.

The reasons for the limited collaboration are multifaceted. Clinicians face constraints on their research time due to increased revenue requirements from hospitals and declining financial support. Additionally, scientists are increasingly pushed towards publication-generating work to maintain funding and advance their careers, often shifting away from time- and resource-intensive translational research [21, 35]. Remarkably, however, it is precisely this cooperation between different interest groups (i.e., universities, clinics, manufacturing companies and regulatory authorities) that is crucial in developing and implementing products [64]. Especially in academic centres that do not have dedicated infrastructures for translational research, a collaboration between preclinical and clinical research groups needs to be strengthened to foster an open culture of joint innovation and knowledge sharing [65, 66]. Thus, given the considerable health and economic challenges, the chances of implementation increase when technologies emerging from academic laboratory environments are more mature and can be readily implemented in quality-controlled, scalable processes and realistically address important specific and potentially multiple clinical indications [36]. It can, therefore, be summarized, based on the results of the survey study, that although the scientists are very experienced in the field of bone defects and biomaterials, the quantity of direct participation in surgical interventions and interdisciplinary case discussions can be increased to intensify corresponding synergies and increase the translational capacity in the field of implant development for bone defect treatment.

Scientists were significantly more inclined to express the opinion that autologous bone grafts can be superseded by bone graft substitutes in the future, expressing optimism about the promising development of these substitutes. This sentiment may stem from the extensive preclinical and experimental (in vitro and in vivo) research on bone substitutes, including allografts and synthetic bone substitutes, for treating bone defects [67]. However, of the countless studies that have aimed in this direction, only a handful have made the leap from bench to bedside, while most have not succeeded in routine clinical implementation [11, 68]. Please note, the results of our study might suggest that surgeons use fewer bone graft substitutes than autologous bone grafts, but this is not what we investigated. Rather, we asked whether bone graft substitutes will replace autologous bone grafts in the future or whether their further development is considered promising. One explanation why surgeons are more reserved in their assessment of bone graft substitutes could be that there is no international consensus on definitions, reliable diagnostic principles, and best practices for the surgical treatment of larger (segmental) bone defects [21, 69, 70]. Noteworthy, from 2008 to 2018, for example, the total number of procedures in Germany in which autografts, allografts, and bone substitutes were used to reconstruct bone defects in the extremities and pelvis increased (2008: 86,294 procedures; 2018: 99,863 procedures, + 15.7%) [71]. The same study also described an increase in the prevalence of the use of bone graft substitutes over the 11-year period, while the absolute numbers of autologous bone grafts decreased (2008: *n* = 63,929, 2018: *n* = 54,784, − 14.3%) [71]. Due to the homogeneous distribution of participants (e.g. 74.5% from Germany), with high numbers from a few countries of origin, conducting a subgroup analysis based on individual countries is not meaningful for the present study. In conclusion, as described above for the German context and thus reflecting the majority of participants in this study, the relative use of bone graft substitutes is increasing compared to autologous bone grafts [71]. However, in order to achieve a more cost-efficient and productive path forward for translation in the future and to support the industry-academia collaborations in their research developments knowingly, a multimodal interdisciplinary international consensus on definitions, reliable diagnostic principles, and best practices for the surgical treatment of larger (segmental) bone defects is particularly needed [21, 72].

Beyond the individual smaller trends described above, market analyses clearly forecast a significant global increase in the compound annual growth rate (CAGR) for autologous bone grafts and bone graft substitutes [71, 73]. This makes it clear that there is an urgent need to enhance the development of bone graft substitutes through direct discourse between surgeons and scientists [74, 75], particularly given the limited economic resources in the healthcare sector. Various allografts and synthetic bone substitutes entail significant costs for the healthcare system due to their costly development and manufacturing processes. For instance, for the use of the allograft DBM (DBM Grafton®, Osteotech Inc, Eatontown, NJ, USA), the costs for augmentation of the bone defect site are higher compared to autologous bone graft (calculated difference: 160 EUR/case, whereby an extended operation time for bone harvesting of autologous bone grafts with associated costs of EUR 213 [76] has already been taken into account [77]). In contrast, no significant differences between the two groups were described concerning complications (*p* = 0.146) or bone healing (*p* = 0.146) [77]. The significant costs associated with autologous bone graft harvesting should not be underestimated. A consecutive series of 50 operations with bone graft harvesting from the iliac crest resulted in total costs of EUR 213 per case (with a mean operation time of 26.3 min) in a German university hospital [76] and USD 4154 (approx. EUR 3435) in a US hospital [78]. This significant difference is likely attributed to the different perspectives on costs, their calculations, and the different healthcare systems. Thus, based on the average positive experience with allografts (e.g. DBM), it is understandable that scientists consider these alternatives and their current developments to be promising. Still, surgeons from the various healthcare systems (and, in particular, the associated costs of these treatment methods) continue to consider the gold standard of autologous bone grafts as the primary treatment option. This sentiment is understandable since most of the surgeons in the study practice in Germany, and the costs of bone graft harvesting there are lower in international comparisons [78]. Interestingly, recent data also reveals that patients prefer autologous sources of cells over pooled cells and blood from different individuals in conjunction with a biodegradable biomaterial to achieve bone regeneration [79]. In line with these findings, there have recently been increased developments by industry, surgeons, and scientists in harvesting autologous bone cells and bone graft tissue [80–82].

**Fig. 6:**
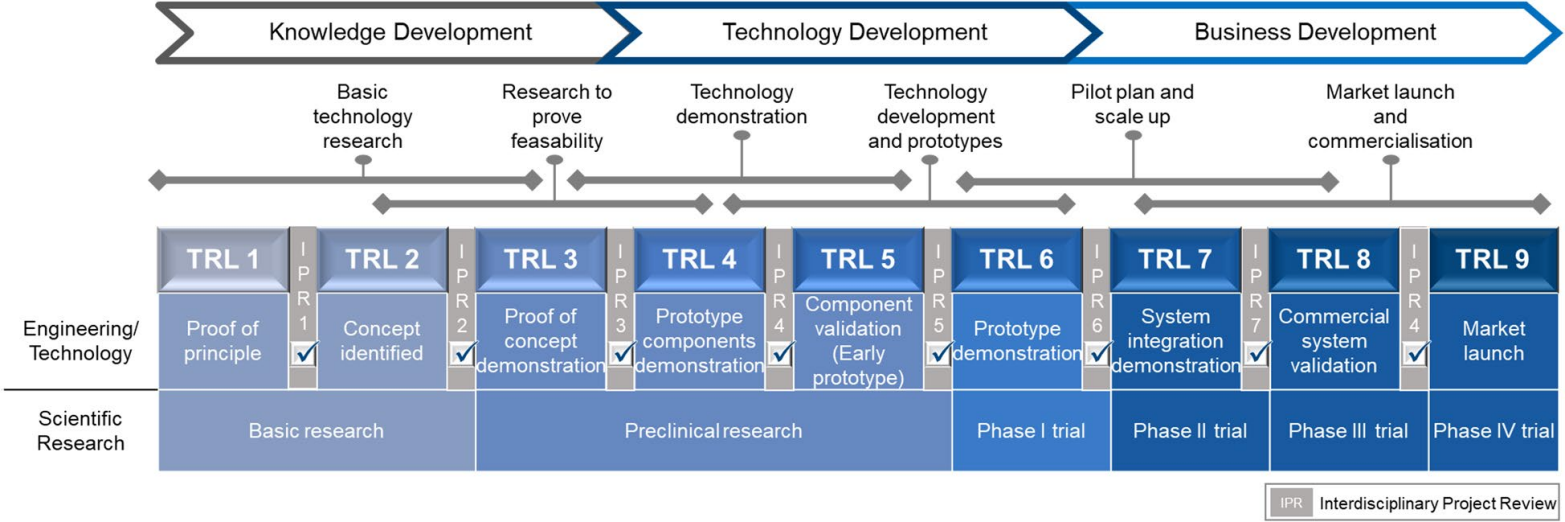
Checklist to ensure the interdisciplinary approach and clinical relevance of medical devices at key points in the product development cycle. Figure adapted from Refs [86, 87]

According to the authors, the different assessments of surgeons and scientists regarding the significance of autologous bone grafts and bone substitute products are also of particular economic and strategic relevance. When asked, for example, whether autologous bone grafts will be replaced by bone substitutes in the future, surgeons were on average 16.9% more likely to deem that this will not be the case. This finding is highly relevant for academic institutions and especially for industry. This means that it may make strategic sense to improve the harvesting of autologous bone grafts to achieve less harvesting morbidity on the one hand and higher quality bone grafts on the other. This is also consistent with recent literature highlighting the need for new technology to replace the current gold standard for harvesting larger volumes of autologous bone graft [6]. According to the authors, a further impetus of the present study is that surgeons were, on average, 13.9% less likely to agree that the current development of bone graft substitutes is promising. This is also reflected in the current literature, which clarifies that no ideal bone substitute product has yet been developed [83]. Significant costs of up to USD 150 million and a development phase of 6–8 years are incurred per product [84], which are invested in relevant cases without being implemented in clinical practice. For future efforts by the medical technology industry regarding research into new bone graft harvesting devices and the development and manufacturing of bone replacement products, it can be summarized that significant development costs could be saved through closer collaboration between surgeons and scientists, moderated by the respective manufacturing companies at the earliest possible product development phase. Moreover, translational research encompasses the iterative process wherein not only do basic scientific findings influence clinical applications, but clinical needs and observations also shape the focus of basic research [85]. Hence why the classification into so-called “Technology Readiness Levels (TRL)” serves to objectively evaluate the maturity of a new technology across its development phases leading up to market launch (Fig. 6).

In line with our findings, there is uncertainty surrounding how recent advances in 3D printing in healthcare will affect standard patient care [88]. Some authors claim that 3D printing will revolutionize healthcare [89, 90], while others argue that caution is warranted as there is little rigorous testing and long-term data on customized 3D-printed products [91]. Indeed, to date, there is no specifically dedicated legal framework for 3D printing medical devices and implants in any jurisdiction [88]. This does not mean that 3D-printed medical devices are unregulated, as the regulations for medical devices are technology-independent, meaning they do not differentiate according to how a product is manufactured. Therefore, the comprehensive existing regulations for medical devices are, in principle, also applicable to 3D-printed medical devices [88]. In the USA, for example, the clinical use of 3D-printed orthopaedic devices is undoubtedly increasing, and the FDA strictly regulates the companies and their products. Therefore, more properly conducted preclinical and clinical studies with sufficiently large sample sizes are needed, particularly in the case of bioresorbable scaffolds for the treatment of large bone defects, before claims of therapeutic efficacy can be accepted and requirements of regulatory authorities for its routine clinical implementation are fulfilled [92]. These findings are also reflected in the results of the present survey study. Both surgeons and scientists consider the need for preclinical (large) animal studies and clinical studies to be very great before 3D-printed scaffolds can be used regularly for bone regeneration.

Surgeons expressed slightly less urgency than scientists on the need for large animal studies. This can also be confirmed in the current literature, which makes it clear that surgeons must demonstrate safety and clinical added value, especially in preparation for human studies [21, 93]. In line with the relevant specialist literature [94, 95], this survey study found that clinical studies play an increasingly central role in the age of evidence-based medicine. Indeed, it is appropriate that diagnostic procedures and non-pharmacological therapies, including therapies with bone substitutes and bone scaffolds, also undergo a needs and risk assessment according to the criteria of evidence-based medicine, similar to the randomized controlled phase III trials required to approve new drugs [96]. The future of clinical research—and, therefore, the future of medical decision-making—will rely heavily on prospective clinical trials [97]. In conjunction with scientists experienced in bone scaffolds, specialist groups active in clinical practice recognize the necessity for 3D printing biodegradable implants such as scaffolds in the future. This recognition is fuelled by promising initial clinical experiences, prompting the initiation of prospective clinical studies [1, 27, 98, 99].

Integrating an independent legal component into the implementation process at an early stage is crucial. The surgeons and scientists emphasized the significant uncertainty and lack of clarity regarding legal requirements. Consistent with our observations, some stakeholders often cite the “perceived” lack of or inadequate regulation as an obstacle to the widespread adoption of bone graft substitutes and 3D-printed medical devices in clinical use. For example, medical devices successfully transferred into clinical practice not only due to a precise understanding of the mechanism of action of their various components but, above all, to compliance with the regulatory framework [11]. The legal uncertainty was clearly reflected in the survey study results, in which more medico-legal clarity is seen as necessary before 3D-printed medical devices are used for standardized bone defect treatment.

Furthermore, over a third of the participants needed to be made aware of the relevant legislation and laws in these cases. It should be emphasized that Regulation (EU) 2017/745, commonly referred to as the “Medical Devices Regulation” (MDR) came into full force for the medical device sector on May 26, 2021, and will soon achieve more standardization at the EU level. Nonetheless, the survey study results indicate a greater need to communicate the new legal regulations within the community of surgeons and scientists and to discuss their consequences for everyday practice. Learning from real-world experiences, such as witnessing the use of the new technology online or via video link, is consistent with the findings from the literature that peer group learning (“clinical champions”) is most effective compared to traditional methods like reading about procedures [100–102]. It is important to note that no biomaterial can completely replace the body’s bone, making it difficult to choose the best replacement [103]. When selecting a material, factors such as tissue survival, size of the bone defect, shape of the graft, biomechanical properties, ease of use, cost, ethical considerations, and potential complications must be taken into account, which may negatively impact the opinion of clinicians on the value and promise of bone graft substitutes [81]. Furthermore, when addressing the controversy surrounding bone graft substitutes, it is vital to consider the challenges associated with the growth factors bone morphogenetic proteins (BMPs), which have contributed to relevant uncertainty regarding the use of synthetic products for bone healing over the last two decades; please refer to further in-depth literature [81]. Notably, the biological and mechanical properties of the bone graft substitute required to achieve bone regeneration successfully should be evaluated in each clinical case, which, in turn, because of its high complexity, it is essential to take an interdisciplinary approach [21].

The global market for bone grafts and substitutes is expected to grow significantly due to an increasing prevalence of orthopaedic disorders and a rising aging population, which drives the demand for bone repair solutions [6, 80]. Additionally, advancements in biomaterials and minimally invasive techniques are fostering the adoption of innovative bone graft substitutes [81, 104, 105]. As the global market for bone grafts and substitutes is set to grow significantly, increased interactions, and regular interdisciplinary (consensus) meetings between surgeons, scientists, and representatives of the medical device industry are necessary to avoid malinvestment of (research) funds in projects without practical relevance. Cooperation across disciplines, especially between surgeons and scientists, must therefore be intensified; this credo is not new and has been called for by individual key figures for at least 75 years [106]. Interdisciplinary collaboration entails more than just regular exchange; it also involves immersing oneself in the everyday life of other disciplines to learn their “language”, which is crucial for fostering high-quality interdisciplinary exchange [85, 106]. Clinicians bring a unique perspective to basic science focusing on biomedical research, deriving focus and motivation from their experience of caring for patients [107]. Becoming a “master” in another discipline is not necessarily required, as noted by Goethe: “Anyone who tries to make music with inadequate talent will of course never become a master, but he will learn to recognize and appreciate what the master has done” (own translation) [108]. Along this continuum from bedside to lab to bedside live the surgeon-scientist and the applied scientist who need more overlap and integration between the “two cultures” Snow described over 60 years ago. The discrepancies in the evaluation of biomaterials for bone regeneration between surgeons and scientists that emerged in this survey underscore the fact that not only support and understanding are needed, but also an infrastructure capable of creating a favourable and productive environment for the success of training this new generation of surgeon-scientists and applied scientists who are proactively engaged in multidisciplinary, inter-institutional, and entrepreneurial collaboration [85].

We note limitations to this study. Strong support from surgical and biomaterials and tissue engineering societies based in Germany resulted in a disproportionately high number of survey participants from Germany. Notably, it is conceivable that religious beliefs and ethical considerations for the use of (allogeneic) bone grafts vary in different regions and cultures, e.g. reservations about the use of products derived from deceased donors or animals [109–111]. Therefore, future studies may benefit from focusing on a more heterogeneous composition of countries of origin, including a more excellent representation from countries of the Global South, to increase (cultural and economic) diversity and the applicability of the results to individual country-specific healthcare systems. Furthermore, numerous and complex steps cost precious time and money in the development and commercialization of medical products. In this study, we have focused on the factors that influence the different perceptions of surgeons and scientists. However, it should be noted that there are other factors affecting successful research and implementation of novel technology such as varying monetary incentives in individual healthcare system and the slow process from (time lag) for research evidence to reach clinical practice which has frequently been stated to be an average of 17 years [112–114].

Since survey studies with more than 10 questions, as was the case in this survey study, are known to result in hasty question completion by most participants within a short time frame without conscious analytical reasoning (i.e. “intuitive System 1” versus “deliberative System 2”) [115–118], to avoid this, the questionnaire was analysed in advance by experts from the Centre for Behavioural Economics, Society and Technology (BEST centre, QUT, Brisbane, Australia) and, if necessary, redesigned so that it did not contain too many and too complex questions that would lead to a hasty (rather emotion-based) response. In particular, the questions were designed so that, as far as subjectively assessable, no higher degree of abstraction was necessary. The minimum required amount of information was given to maximize the clarity of the respondents’ assessments of the survey questions [119]. Nevertheless, the effects of unbalanced System 1 / System 2 involvement cannot be ruled out.

## Conclusions

To conclude, understanding the decision-making processes of surgeons and scientists regarding current options and future possibilities for treating bone defects is crucial for (cost-) effective product development, especially considering that the total cost of bone reconstruction worldwide is USD 1700 billion [120] and the global market for bone grafts and substitutes is expected to grow at a CAGR of 5.8% from 2021 to 2028 [74]. Therefore, this prospective survey to assess the development of future biomaterials for bone defects was a suitable tool to collect relevant information regarding the *status quo*, for strategic foresight and policy making, and to learn more about the future developments, opinions, and behaviours of surgeons and scientists. Our results suggest that improving the relationship between (fundamental) research, clinical, and industry stakeholders, for example through closer exchange or regular consensus meetings, would benefit all parties involved in enhancing the development of biomaterials and, ultimately, the care of patients with bone defects.

The report by Madry et al. [121] from the 2013 symposium “Where Science meets Clinics”, hosted by the AO Foundation (https://www.aofoundation.org), already conveyed the perspectives of the different stakeholders of the clinical implementation process of orthopaedic regenerative bone tissue engineering, including academic scientists, clinicians, industry and regulators, and emphasized the need for a “translational research environment” that has effective communication between all stakeholders throughout the project. Moreover, as suggested by Hollister and Murphy [36] and more recently by Duda et al. [46], research project designs must target specific clinical indications and include the regulatory approval pathway, implementation of quality systems for the proposed technology, and the potential market, early in the study design. It could be described as removing philosophical barriers to recognize the unique needs of translational science and engineering and establishing a multidisciplinary approach and cross-laboratory visits, including operating rooms, especially involving clinicians and biomedical scientists to promote understanding of key elements of their respective fields [1, 21]. For tangible and immediate action, an interdisciplinary consensus meeting is urgently needed to address prospective investigations of bone substitutes and (3D-printed) bone scaffolds for the treatment of bone defects and to discuss specific surgical indications, (pre-)clinical studies to be performed, relevant medico-legal aspects, and clinical implementation strategies.

## Supplementary Information

Supplementary Material 1.

## Data Availability

The paper contains all data associated with this study. Upon a reasonable request, Stephen Whyte can provide the raw survey data under a material transfer agreement with the Queensland University of Technology.

## Abbreviations

3D printing: Three-dimensional printing
BEST centre: Centre for behavioural economics, society and technology
CAGR: Compound annual growth rate
DAC: Development assistance committee
DBM: Demineralized bone matrix
BMP: Bone morphogenetic protein
EU: European Union
FDA: Food and drug administration
HA: Hydroxyapatite
MDR: Medical device regulation
mPCL-TCP: Medical-grade polycaprolactone and tricalcium phosphate
QUT: Queensland University of Technology
OECD: Organization for economic co-operation and development
OSF: Open science framework
SD: Standard deviation
SGBR: Scaffold-guided bone regeneration
TCP: Tricalcium phosphate
TRL: Technology readiness level
USA: United States of America
