# Supplementary material for: Lost in translation: the lack of agreement between surgeons and scientists regarding biomaterials research and innovation for treating bone defects
Source: BMC Med. 2024 Nov 6;22:517. doi: 10.1186/s12916-024-03734-z (PMC11542434; doi:10.1186/s12916-024-03734-z)
Supplement: Supplementary file 1 — Supplementary Material 1. [file 12916_2024_3734_MOESM1_ESM.docx]

Supplement

Lost in translation: The lack of agreement between surgeons and scientists regarding biomaterials research and innovation for treating bone defects

**Authors:** Markus Laubach ^1,2,*^; Stephen Whyte ^3,4,5,6,*^; Ho Fai Chan ^3,4,6^; Tina Frankenbach-Désor ^1^; Susanne Mayer-Wagner ^1^; Frank Hildebrand ^7^; Boris M. Holzapfel ^1^; Ulrich Kneser ^8^; Uwe Dulleck ^3,4,5,6,9^; Dietmar W. Hutmacher ^2,4,5,10,11,*^

### Affiliations:

^1^ Department of Orthopaedics and Trauma Surgery, Musculoskeletal University Center Munich (MUM), LMU University Hospital; LMU Munich, Munich, Germany.

^2^ Australian Research Council (ARC) Training Centre for Multiscale 3D Imaging, Modelling, and Manufacturing (M3D Innovation), Queensland University of Technology; Brisbane, QLD 4000, Australia.

^3^ School of Economics and Finance, Queensland University of Technology (QUT); 2 George St, Brisbane, QLD 4001, Australia.

^4^ Centre for Behavioural Economics, Society & Technology (BEST), Queensland University of Technology (QUT); Brisbane, QLD 4001, Australia.

^5^ ARC Training Centre for Cell and Tissue Engineering Technologies, Queensland University of Technology (QUT); Brisbane, QLD 4059, Australia.

^6^ ARC Training Centre for Behavioural Insights for Technology Adoption, Queensland University of Technology (QUT); Brisbane, QLD 4001, Australia.

^7^ Department of Orthopaedics, Trauma and Reconstructive Surgery, RWTH Aachen University Hospital; Pauwelsstraße 30, 52074 Aachen, Germany

^8^ Department of Hand, Plastic, and Reconstructive Surgery, Microsurgery, Burn Center, BG Trauma Centre Ludwigshafen, University of Heidelberg; Ludwigshafen, Germany.

^9^ Faculty of Business Government and Law, University of Canberra, Australia

^10^ ARC Training Centre in Additive Biomanufacturing, Queensland University of Technology; Brisbane, QLD 4059, Australia.

^11^ Max Planck Queensland Centre (MPQC) for the Materials Science of Extracellular Matrices, Queensland University of Technology; Brisbane, QLD 4000, Australia

^*^Corresponding authors. Markus.Laubach@med.uni-muenchen.de, sg.whyte@qut.edu.au, dietmar.hutmacher@qut.edu.au

# Supplement 1

## Complete questionnaire of the web-based survey.

# First (landing) page


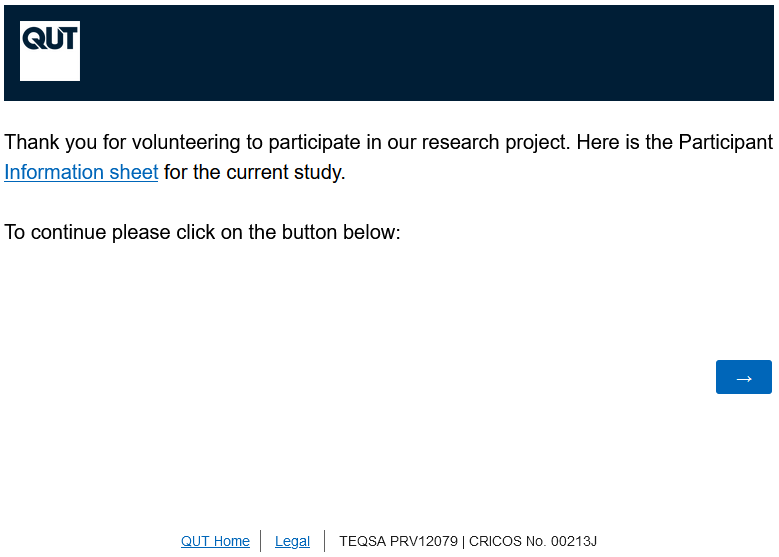


# Page 2


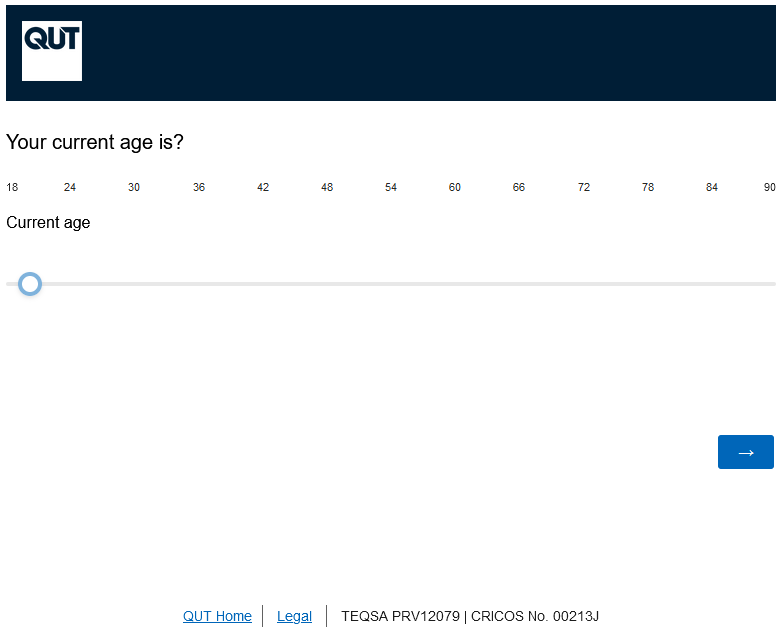


# Page 3


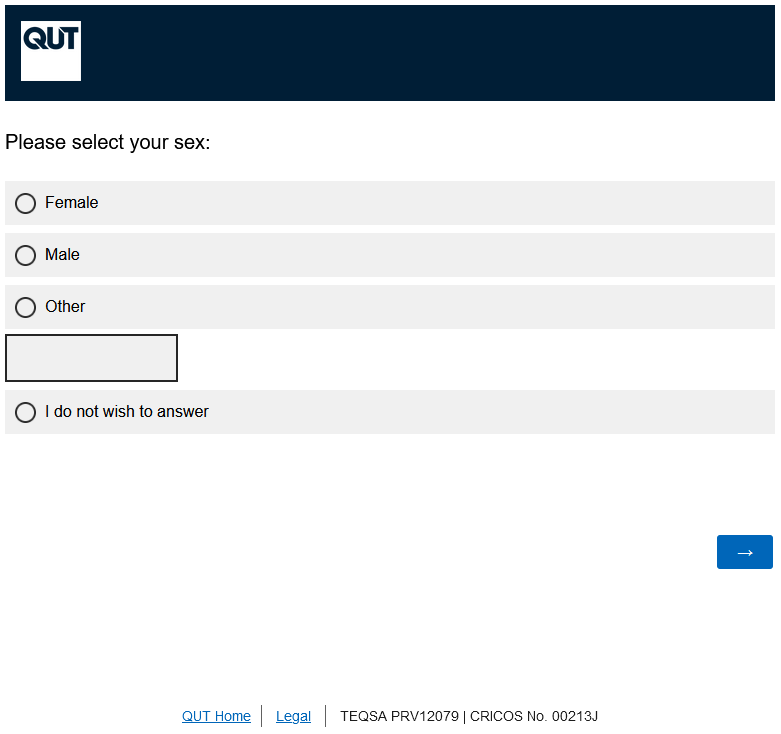


# Page 4


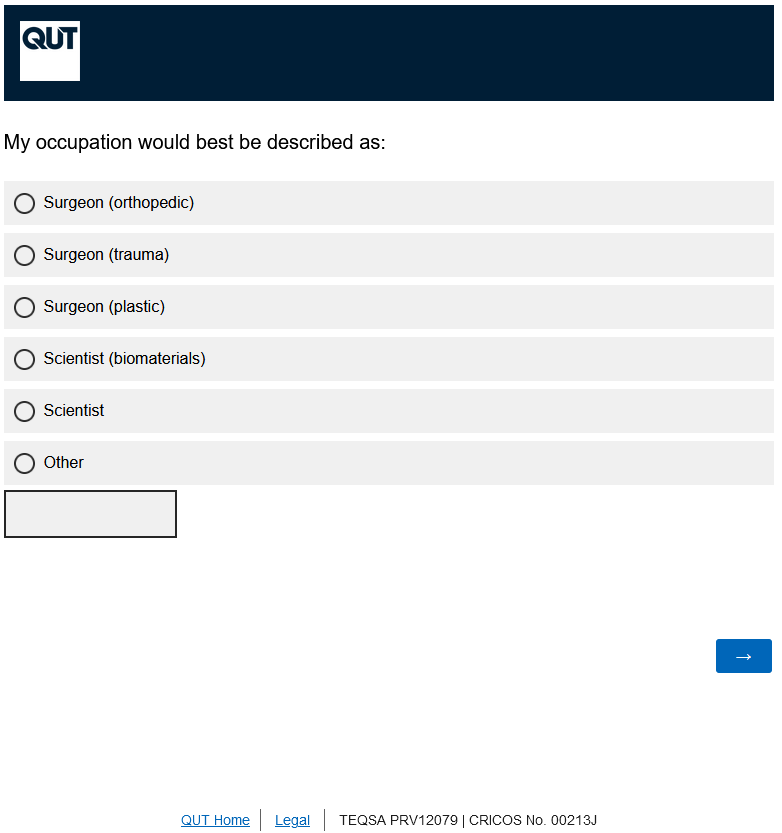


# Page 5


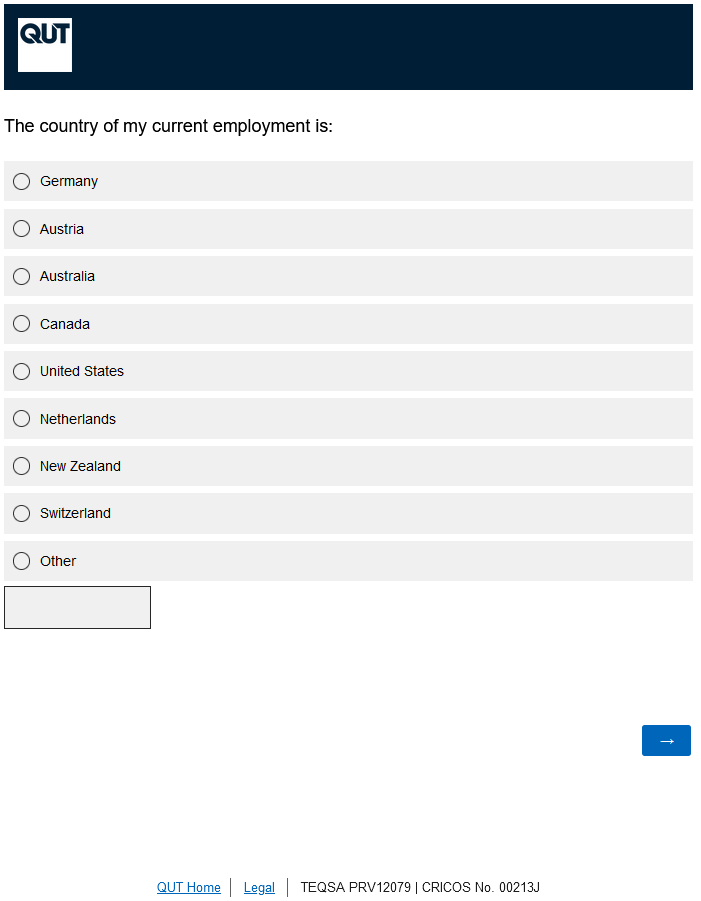


# Page 6


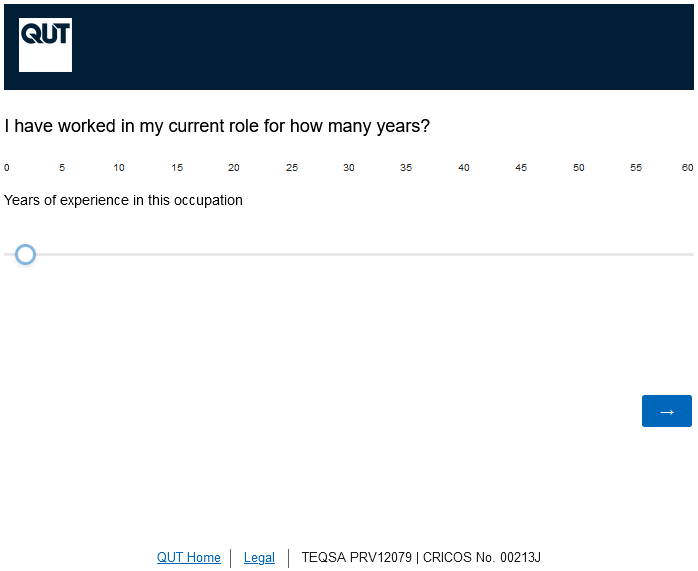


# Page 7


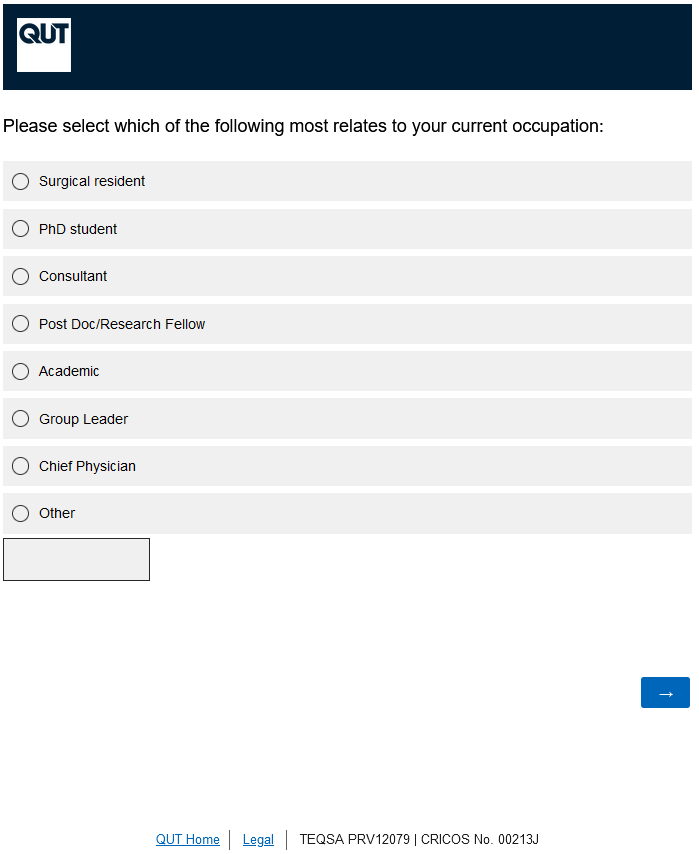


# Page 8


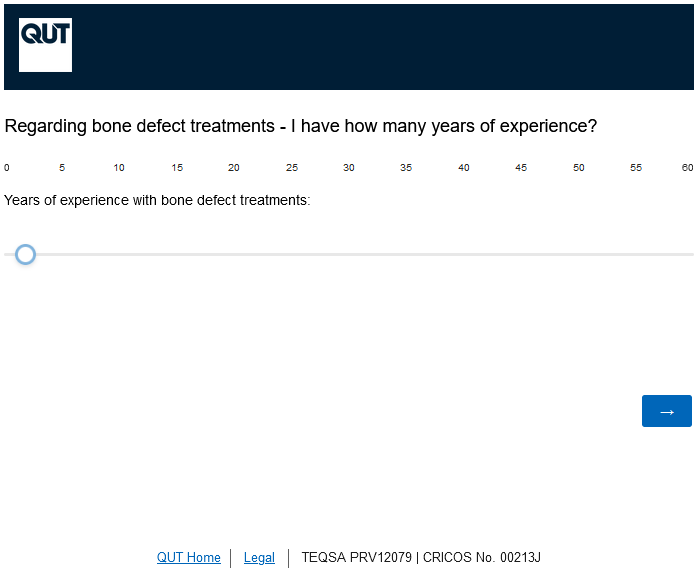


# Page 9


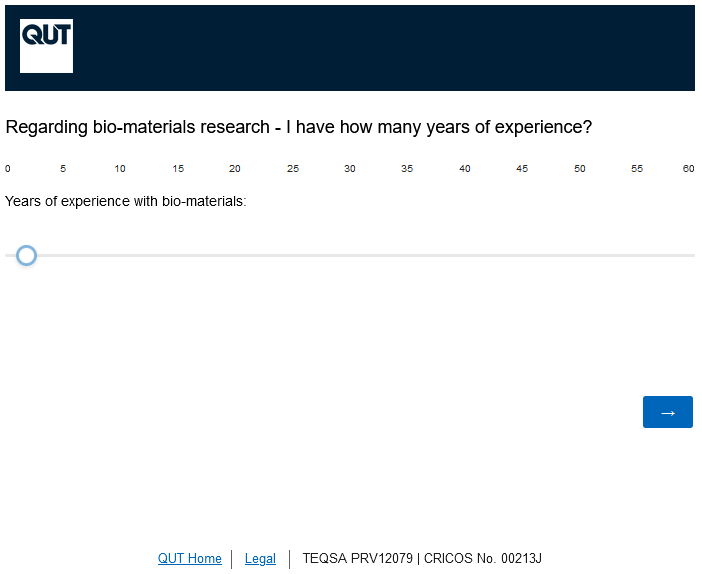


# Page 10


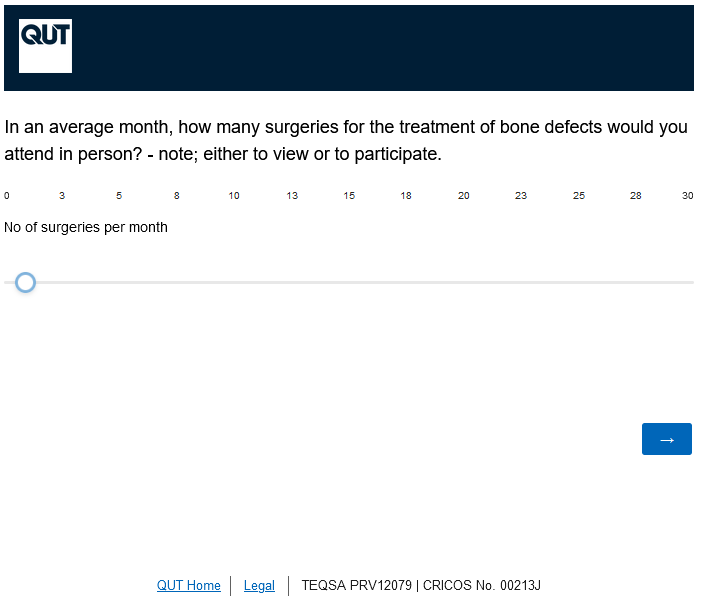


# Page 11


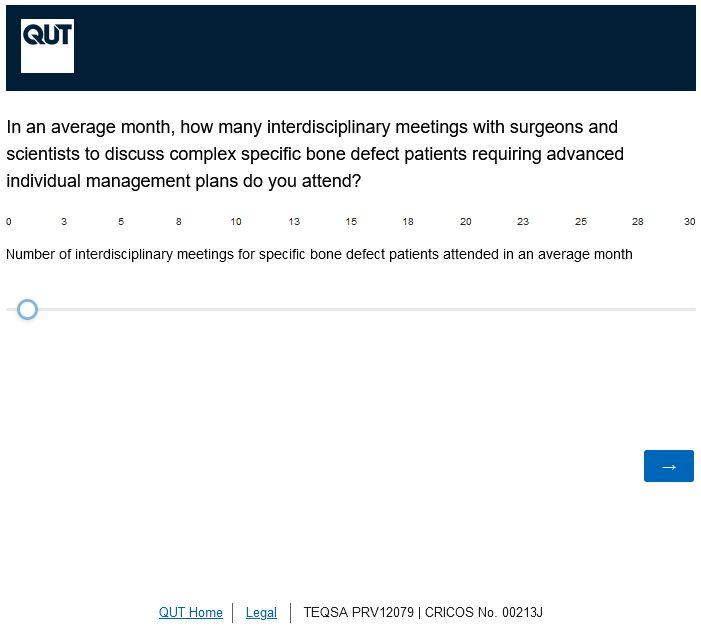


# Page 12


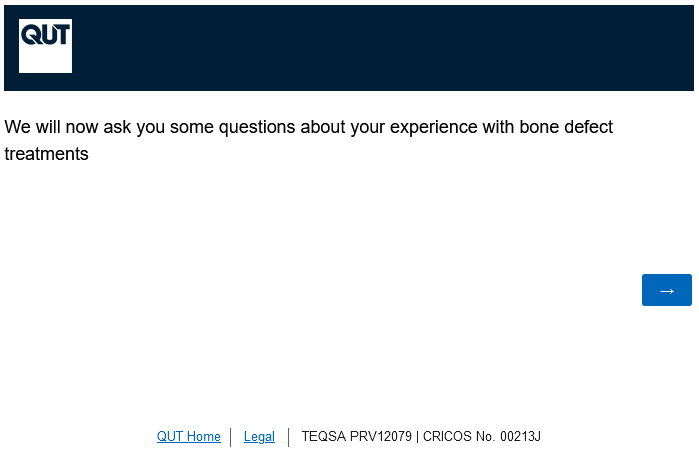


# Page 13


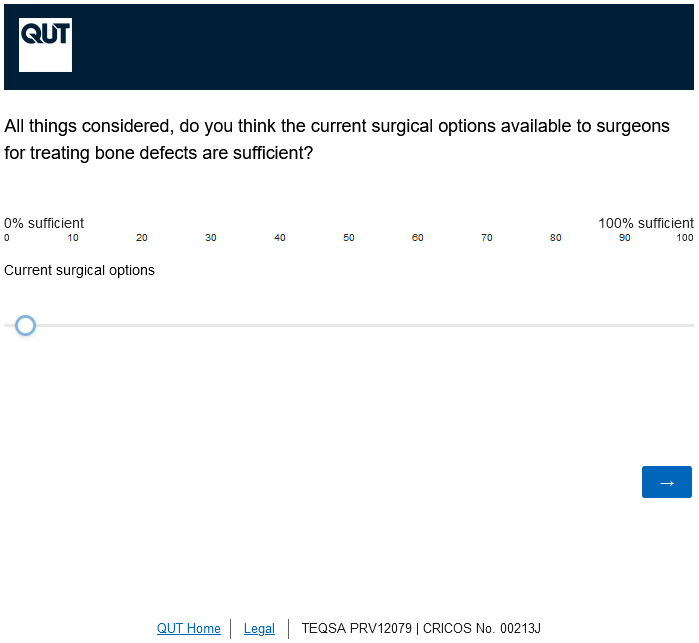


# Page 14


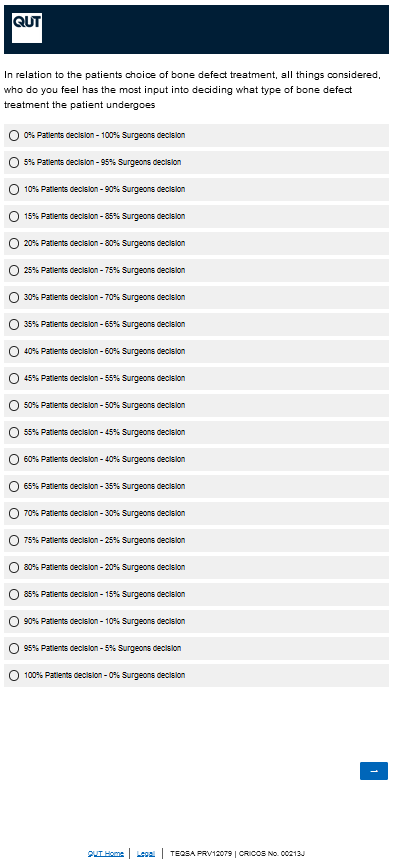


# Page 15


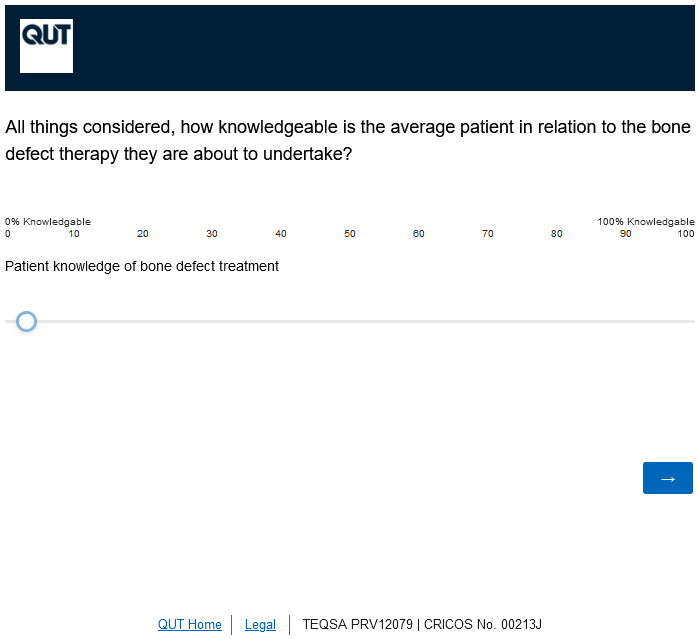


# Page 16


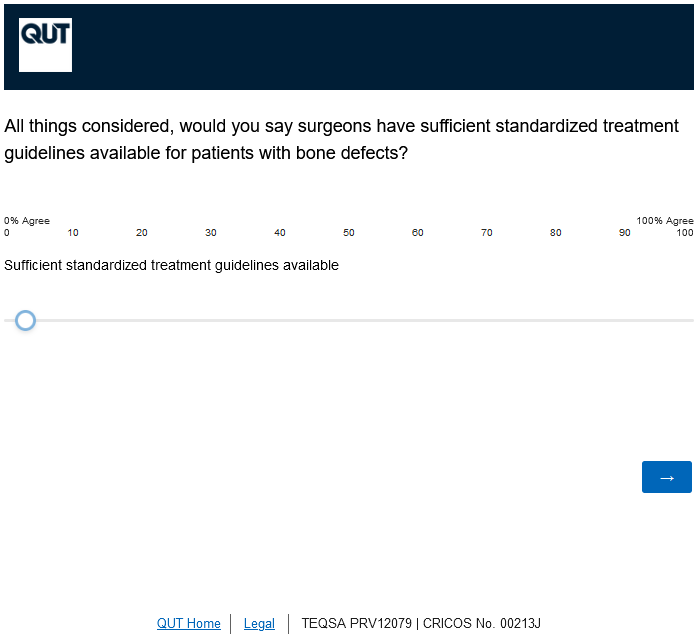


# Page 17


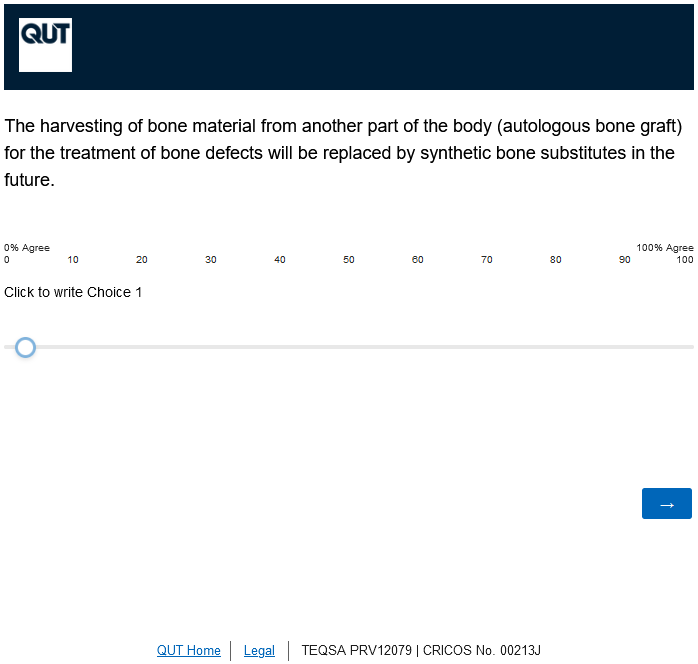


# Page 18


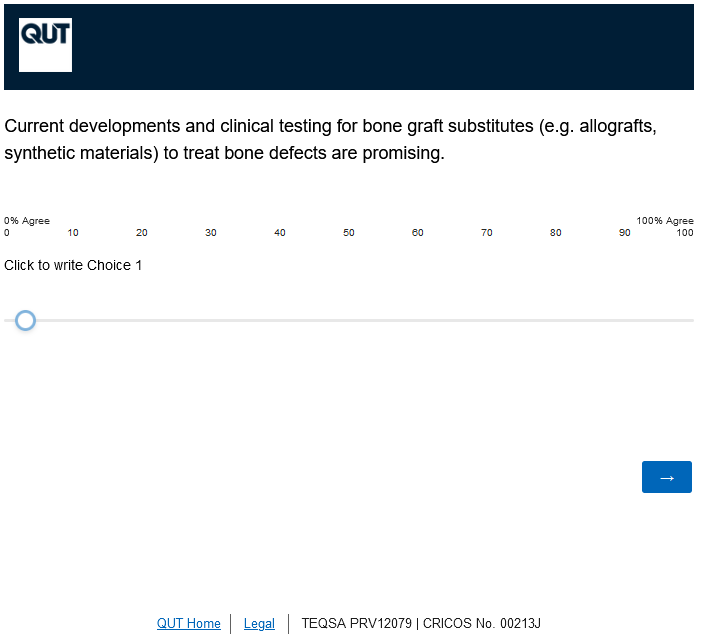


# Page 19


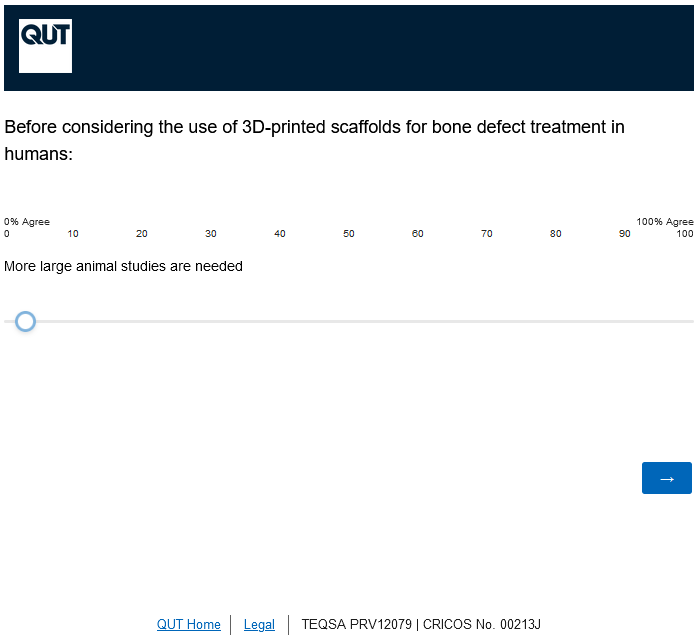


# Page 20


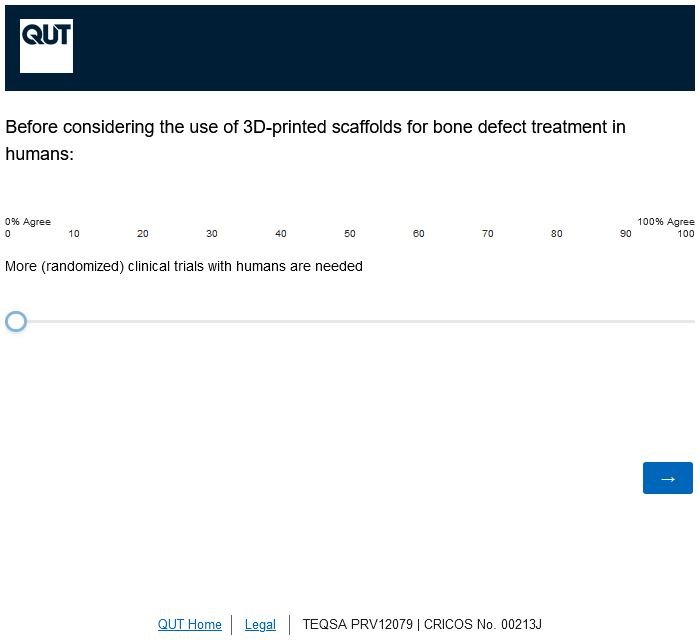


# Page 21


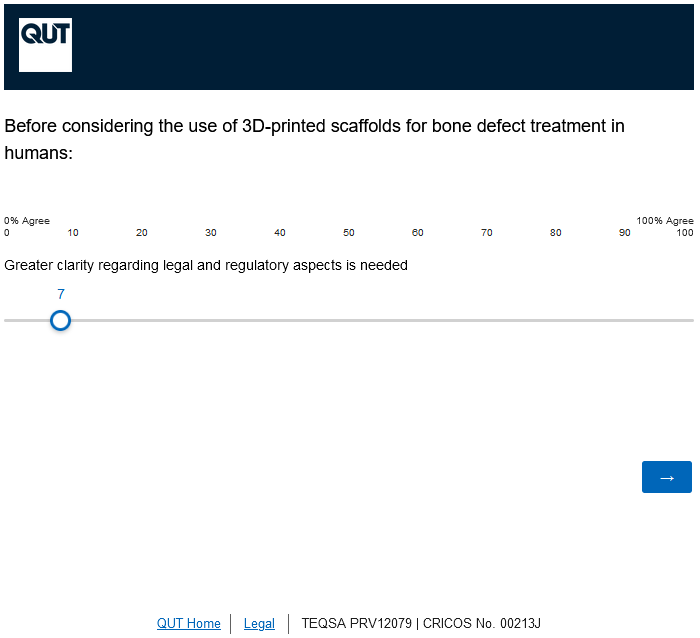


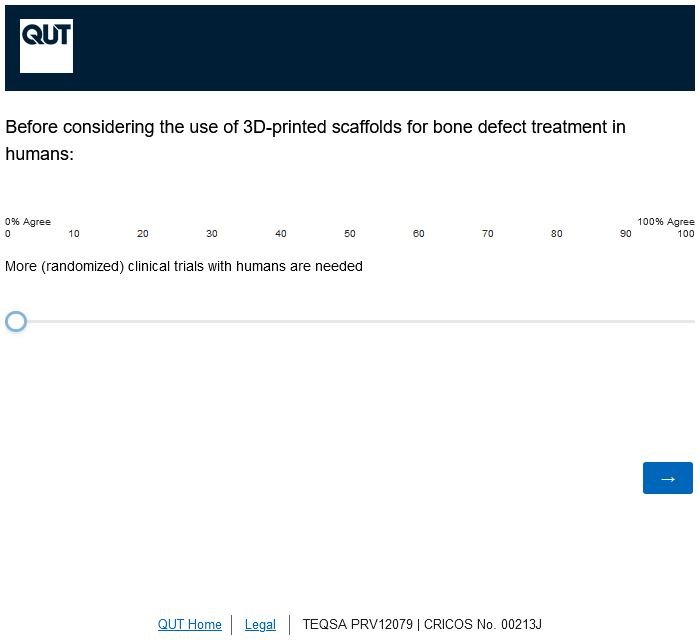


# Page 22


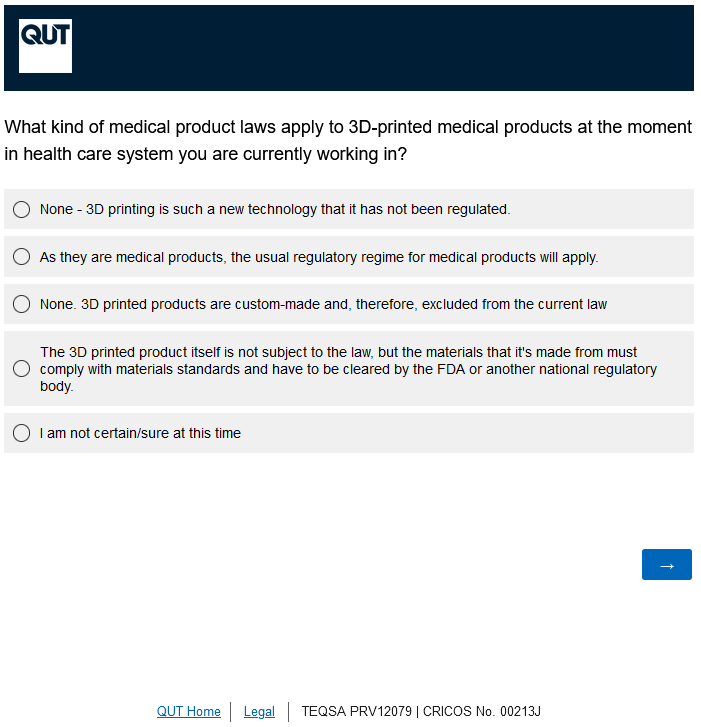


# Page 23


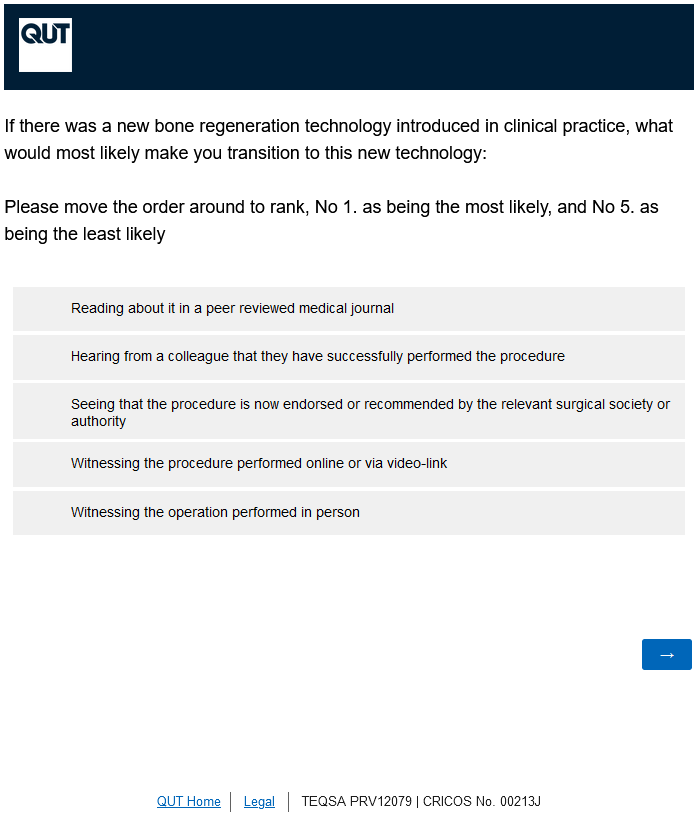


# Page 24


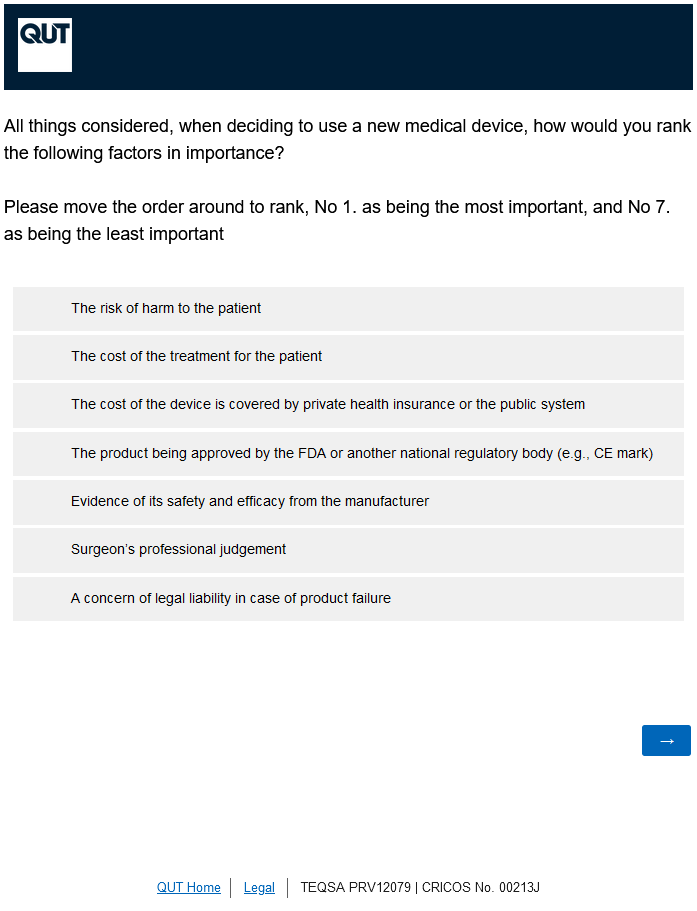


# Page 25


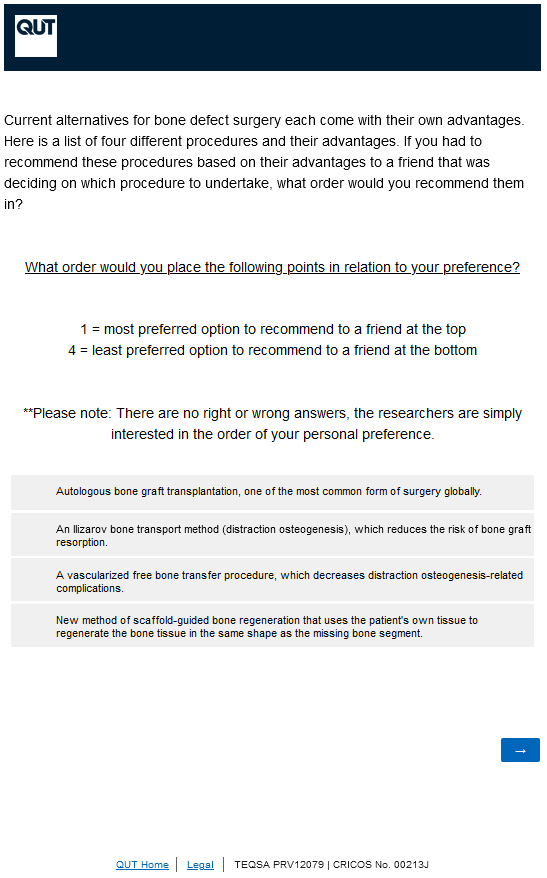


# Page 26


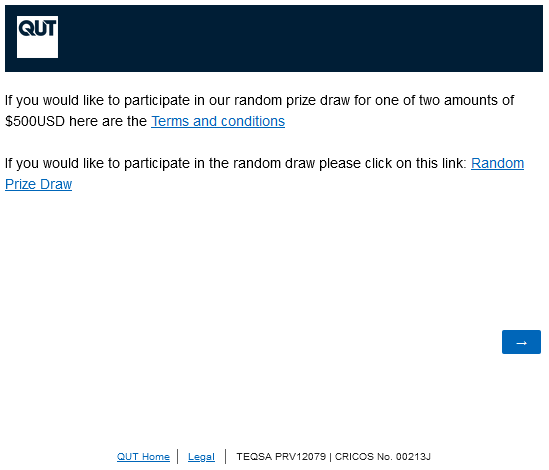


# Page 27


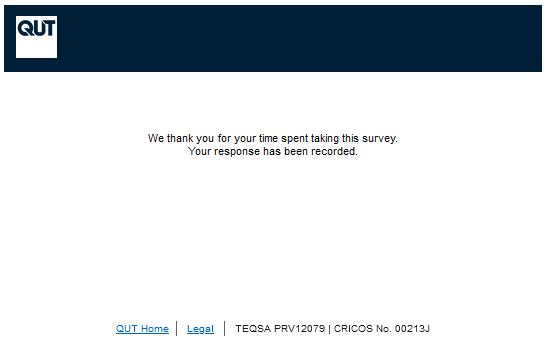


# Supplement 2

## Number of responses per survey question.

|  | **Number of responses** | |
| --- | --- | --- |
| **Survey question** | **Surgeons (N)** | **Scientists (N)** |
| Regarding bone defect treatments – I have how many years of experience? | 319 | 90 |
| Regarding bio-materials research – I have how many years of experience? | 316 | 87 |
| In an average month, how many surgeries for the treatment of bone defects would you attend in person? – note; either to view or to participate. | 292 | 85 |
| In an average month, how many interdisciplinary meetings with surgeons and scientists to discuss complex specific bone defect patients requiring advanced individual management plans do you attend? | 282 | 81 |
| All things considered, do you think the current surgical options available to surgeons for treating bone defects are sufficient? | 259 | 69 |
| All things considered, would you say surgeons have sufficient standardized treatment guidelines available for patients with bone defects? | 252 | 68 |
| The harvesting of bone material from another part of the body (autologous bone graft) for the treatment of bone defects will be replaced by synthetic bone substitutes in the future. | 247 | 65 |
| Current developments and clinical testing for bone graft substitutes (e.g. allografts, synthetic materials) to treat bone defects are promising. | 247 | 65 |
| Before considering the use of 3D-printed scaffolds for bone defect treatment in humans: More large animal studies are needed | 249 | 66 |
| Before considering the use of 3D-printed scaffolds for bone defect treatment in humans: More (randomized) clinical trials with humans are needed | 248 | 66 |
| Before considering the use of 3D-printed scaffolds for bone defect treatment in humans: Greater clarity regarding legal and regulatory aspects is needed | 246 | 66 |
| What kind of medical product laws apply to 3D-printed medical products at the moment in health care system you are currently working in? | 241 | 66 |
| If there was a new bone regeneration technology introduced in clinical practice, what would most likely make you transition to this new technology? | 210 | 58 |
